# Phosphorylation remodels the mitotic centrosome matrix to generate bipartite γ-tubulin complex docking sites

**DOI:** 10.1126/sciadv.aed6539

**Published:** 2026-05-27

**Authors:** Midori Ohta, Orie Arakawa, Yajie Gu, Wanying Tian, Kevin D. Corbett, Arshad Desai, Karen Oegema

**Affiliations:** ^1^ Okinawa Institute of Science and Technology Graduate University, Okinawa, Japan.; ^2^ Department of Cellular and Molecular Medicine, University of California, San Diego, La Jolla, CA 92093, USA.; ^3^ Department of Cell and Developmental Biology, School of Biological Sciences, University of California, San Diego, La Jolla, CA 92093, USA.; ^4^ Department of Molecular Biology, School of Biological Sciences, University of California, San Diego, La Jolla, CA 92093, USA.

## Abstract

Mitotic centrosomes consist of centrioles surrounded by a proteinaceous matrix that docks and activates γ-tubulin complexes (γTuCs) to nucleate microtubules for spindle assembly. During mitotic entry, phosphorylation at centrosomes remodels CDK5 regulatory subunit associated protein 2 (CDK5RAP2) family matrix proteins to generate γTuC docking sites. We address the mechanism of this conversion using *Caenorhabditis elegans* SPindle Defective (SPD-5) as a model. We show that SPD-5 contains two regions, phospho-regulated γTuC binding region 1 (PRGB1) and PRGB2, that are each sufficient for polo-like kinase 1 (PLK1) phosphorylation–regulated γTuC binding. We define key phosphosites in each region and uncover autoinhibition mediated by interactions within and between them. PRGB2 is dimeric and requires γTuCs containing the Mozart family microprotein MZT-1 for binding, whereas PRGB1 is monomeric and binds independently of MZT-1. Our results support a model in which PLK1 phosphorylation induces a conformational change that enables MZT-1–dependent PRGB2 engagement, which in turn relieves PRGB1 inhibition. Such a multistep mechanism would ensure robust spindle assembly by restricting microtubule nucleation in space and time.

## Teaser

Phosphorylation releases multiple layers of inhibition to enable centrosomal microtubule nucleation for spindle assembly.

## INTRODUCTION

Centrosomes consist of a centriolar core surrounded by a pericentriolar material (PCM) matrix that nucleates and anchors microtubules (*1*, *2*). During cell division in metazoans, centrosomes catalyze assembly of the mitotic spindle (*3*–*9*). In preparation for mitosis, the PCM matrix increases in size and microtubule nucleation capacity through a process controlled by polo-like kinase 1 (PLK1) (*10*–*16*). Across systems, the PCM matrix is assembled primarily from CDK5 regulatory subunit associated protein 2 (CDK5RAP2)-family proteins: CDK5RAP2 in humans, centrosomin (Cnn) in *Drosophila*, and SPindle Defective (SPD-5) in *Caenorhabditis elegans* (*17*). In *Drosophila* and *C. elegans*, PCM expansion is driven by PLK1 phosphorylation–dependent self-interaction of these scaffold proteins (*11*, *16*, *18*, *19*). However, how PLK1 phosphorylation remodels the scaffold to generate mitotic microtubule nucleation sites remains unclear.

Complexes containing the specialized tubulin isoform γ-tubulin make a major contribution to microtubule nucleation by mitotic centrosomes (*20*–*23*). γ-Tubulin is incorporated into Y-shaped heterotetramers, termed γ-tubulin small complexes (γTuSCs), which contain two γ-tubulin molecules supported on stalks composed of two related proteins, GCP2 and GCP3. Across systems, γTuSCs can laterally associate to form larger γ-tubulin ring complexes (γTuRCs), either in the cytoplasm or upon recruitment to PCM matrix docking sites (*20*, *21*, *23*). Vertebrates and *Drosophila* have soluble γTuSCs and γTuRCs (*24*–*26*); structural studies have shown that the latter assemble from multiple γTuSC-like units, some of which incorporate γTuRC-specific GCPs (GCP4-6) (*27*–*30*). In sucrose gradients of *C. elegans* embryo extracts, GCP2 and GCP3 have been detected in γTuSC-sized complexes and in larger assemblies (*31*). *C. elegans* lacks GCP4-6, but two *Caenorhabditis*-specific proteins, gamma-tubulin associating protein (GTAP-1) and GTAP-2, have been proposed to substitute for GCP4-6 to promote the formation of larger γ-tubulin–containing complexes (*31*). Because GTAP-1 and GTAP-2 are not essential for centrosomal microtubule nucleation, γTuSCs themselves are likely sufficient for PCM recruitment (*31*).

Work in *Drosophila* suggests two principal pathways for the centrosomal recruitment of γ-tubulin complexes (γTuCs): Spd-2–mediated recruitment of preassembled γTuRCs and recruitment of γTuSCs or γTuRCs by Cnn (*32*–*34*). Humans, *Xenopus*, and *Drosophila* have γTuSCs and γTuRCs (*24*–*26*, *35*, *36*) and likely use both recruitment pathways. In contrast, in *C. elegans*, all recruitment of γ-tubulin to centrosomes requires the CDK5RAP2-like protein SPD-5 (*37*), suggesting that a single recruitment pathway is used. The fact that centrosomal microtubule nucleation is normal in the absence of the candidate γTuRC components GTAP-1 and GTAP-2 (*31*) suggests that SPD-5 can recruit γTuSCs and potentially also γTuRCs.

The nucleating activity of γ-tubulin–containing complexes is thought to be activated upon recruitment to microtubule-organizing centers (MTOCs) such as centrosomes (*21*, *23*). During mitosis in *C. elegans*, PLK1 phosphorylates the SPD-5 N terminus to convert it into a γTuC docking site (*38*). In *Drosophila*, phosphorylation of the N terminus of Cnn has similarly been proposed to generate γTuC docking sites by relieving an autoinhibited state (*39*). Early studies in fission yeast identified a conserved protein sequence shared by γTuC docking proteins that was later named centrosomin motif 1 (CM1) (*40*, *41*). Work in human cells partitioned CM1 into two conserved regions, a stimulatory N-terminal region and an autoinhibitory C-terminal region. The N-terminal portion contains an ~30–amino acid peptide termed γTuRC-mediated nucleation activator (γTuNA) (*42*). In vertebrates, the γTuNA forms a dimeric coiled-coil capable of binding and activating γTuRCs (*42*–*48*). Extension of human CDK5RAP2 fragments beyond the γTuNA to include the second conserved CM1 motif, termed γTuNA inhibitor (γTuNA-In), abolishes interaction with γTuCs (*49*). Centrosomal docking and activation of γTuCs also involves small Mozart family microproteins. MZT-1 is a widely conserved three-α-helix protein required for γTuCs to interact with its docking sites at MTOCs across eukaryotes (*23*, *50*). MZT-1 intercalates with the N-terminal helical domain (NHD) of GCP3 as well as with other γTuRC-specific GCPs (*23*, *30*, *43*, *45*, *51*–*59*). Vertebrates also encode MZT-2, which intercalates into the GCP2 NHD where it forms an interaction surface for the CDK5RAP2 γTuNA coiled-coil; this interaction promotes closure of the γTuRC into an activated conformation (*30*, *45*, *47*, *48*, *58*). Together, these studies support a model in which γTuC docking sites on PCM matrix molecules are autoinhibited until they are activated by phosphorylation-dependent structural remodeling. However, how phosphorylation restructures the N termini of CDK5RAP2-like proteins to enable productive γTuC engagement remains poorly understood.

Here, we use the *C. elegans* PCM matrix protein SPD-5 as a model to determine how phosphorylation converts a CDK5RAP2-family protein into a functional γTuC docking site. We show that the SPD-5 N terminus contains two regions positioned on either side of a γTuNA-In–like inhibitory sequence that are each capable of PLK1 phosphorylation–regulated γTuC binding in vitro and are required for γTuC recruitment in vivo. We term these regions phospho-regulated γTuC binding region 1 (PRGB1) and PRGB2 and show that they are mechanistically distinct in their modes of γTuC engagement. In addition to positive phosphoregulation of each region, we uncover autoinhibitory interactions within and between them. In the context of the intact N terminus, PLK1 phosphorylation induces a conformational change that enables MZT-1–dependent binding of PRGB2 to the γTuC, which in turn allows PRGB1, containing the CM1 region, to bind to the γTuC. Together, our findings reveal a multistep, phosphorylation-driven conversion of a PCM matrix scaffold protein into a bipartite γTuC docking site and provide a mechanistic framework for how centrosomes spatially restrict microtubule nucleation during mitotic entry.

## RESULTS

### SPD-5 fragments exhibit a complex pattern of phosphorylation-controlled binding to γTuCs

**Fig. 1.**
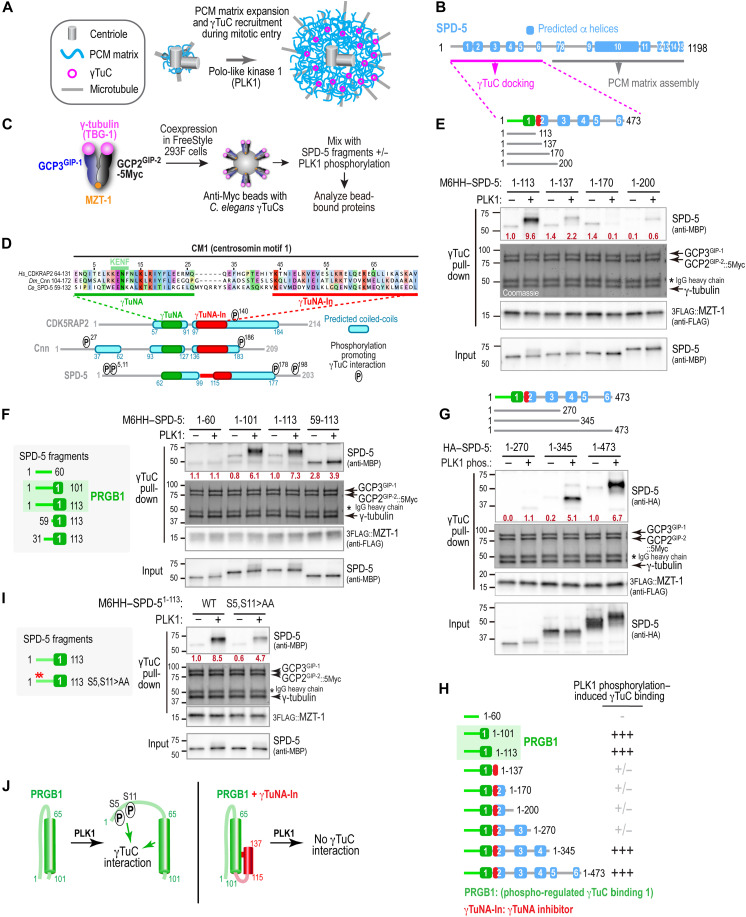
SPD-5 N-terminal fragments exhibit a complex pattern of PLK1 phosphorylation–dependent binding to γTuCs. (**A**) Schematic illustrating PLK1-driven centrosome maturation through phosphorylation of the CDK5RAP2-family protein SPD-5. (**B**) Diagram of SPD-5 showing 15 predicted α helices. Phosphorylation of its C-terminal half promotes matrix assembly, whereas phosphorylation of its N terminus converts it into a γTuC docking site. (**C**) Schematic of the in vitro assay used to assess PLK1-dependent γTuC binding by SPD-5 fragments. γ-tubulin^TBG-1^, GCP2^GIP-2^-5Myc, GCP3^GIP-1^, and MZT-1 are coexpressed in FreeStyle 293F cells, and complexes are immunoisolated onto Myc antibody-coated beads. SPD-5 fragments, purified from bacteria and preincubated with or without PLK1 in the presence of adenosine 5′-triphosphate (ATP), are added to γTuC-coated beads, and bound proteins are analyzed. (**D**) ClustalW alignment of the CM1 region from human CDK5RAP2, *Drosophila* Cnn, and *C. elegans* SPD-5. Schematics of the N termini of the three proteins highlight predicted coiled-coils (PCOILS 21–amino acid window rounded score ≥ 0.5), positions of the sequences aligned with the γTuNA (green) and γTuNA-In (red), and previously identified phosphorylation sites that promote γTuC binding [(*38*, *39*, *76*); SPD-5 S5 and S11 identified here]. (**E** to **G** and **I**) Binding assays performed as in (C) using γTuC-coated beads and MBP-6His-HA (M6HH)–tagged (E, F, and I) or hemagglutinin (HA)–tagged (G) SPD-5 fragments, preincubated with or without PLK1. SPD-5 and MZT-1 were detected by immunoblotting; γ-tubulin, GCP2^GIP-2^, and GCP3^GIP-1^ were visualized by Coomassie staining. Numbers in dark red below the SPD-5 fragment bands indicate band intensity relative to the SPD-5 1–113 (E, F, and I) or 1–473 (G) in the absence of PLK1 phosphorylation. Asterisk indicates the immunoglobulin G (IgG) heavy chain of the anti-Myc antibody. (**H**) Schematic summary of SPD-5 fragments used in (E) to (G) and their PLK1 phosphorylation–induced γTuC-binding. (**J**) Speculative model illustrating how PLK1 phosphorylation affects γTuC binding by fragments containing PRGB1 (left) or PRGB1 plus the γTuNA-In (right).

During mitotic entry in the *C. elegans* embryo, the SPD-5–containing PCM matrix surrounding the centrioles increases five- to 10-fold in size and microtubule nucleating capacity in a process known as centrosome maturation (Fig. 1A) (*37*, *60*, *61*). Centrosome maturation is driven by the mitotic kinase PLK1 (*10*–*16*) and the *C. elegans* homolog of centrosomal protein 192 (CEP192), SPD-2 (*62*, *63*). SPD-2 contains a mitotic docking site for PLK1 and binds SPD-5, thereby delivering PLK1 to the PCM matrix (*64*, *65*). PLK1 phosphorylates the central region of SPD-5 to promote SPD-5–SPD-5 interactions that drive matrix expansion and independently phosphorylates the SPD-5 N terminus to convert it into a γTuC docking site (Fig. 1B) (*16*, *38*).

To understand how PLK1 phosphorylation converts the SPD-5 N terminus into a γTuC docking site, we combined an in vitro reconstitution system with in vivo analysis (*38*). In this reconstitution, the core components of the *C. elegans* γTuC—γ-tubulin^TBG-1^, GCP2^GIP-2^, GCP3^GIP-1^, and the Mozart-family protein MZT-1 (*38*, *66*, *67*)—are coexpressed in FreeStyle 293F human cells and immunopurifed onto beads (Fig. 1C). Because they are not required for centrosomal microtubule nucleation (*31*), the *Caenorhabditis*-specific γTuC-associated proteins GTAP-1 and GTAP-2 are not included in the reconstitution. The ability of bacterially purified SPD-5 fragments to bind γTuC-coated beads in the presence or absence of PLK1 phosphorylation is then assessed. In prior work, we showed that SPD-5 amino acids 1 to 473 exhibits robust PLK1 phosphorylation–dependent binding to γTuC-coated beads and identified two predicted PLK1 phosphorylation sites (T178 and T198) that are important for binding (*38*). However, how phosphorylation at these and additional sites within the SPD-5 N terminus stimulates γTuC binding remains unknown.

Prior work on γTuC docking sites in CDK5RAP2-family proteins has focused on their CM1 regions (*23*, *40*–*44*, *46*–*48*, *58*). Sequence alignments of the SPD-5 CM1-like region with the CM1 regions of human CDK5RAP2 and *Drosophila* Cnn (Fig. 1D) suggested that the *C. elegans* sequence contains two adjacent regions that can be aligned with the previously described γTuNA (amino acids 59 to 84) (*42*) and γTuNA inhibitor (γTuNA-In; amino acids 102 to 132) (*49*) motifs. Notably, the putative SPD-5 γTuNA exhibits divergence at key conserved residues, including the lysine and phenylalanine in the KENF motif (Fig. 1D), which motivated us to functionally test whether this region mediates binding to the γTuC in the in vitro reconstitution assay.

Consistent with the alignment, a short N-terminal fragment of SPD-5 (amino acids 1 to 113) containing the predicted γTuNA—but not the γTuNA-In—bound γTuC when phosphorylated by PLK1 (Fig. 1, E and F). A slightly shorter fragment SPD-5 (amino acids 1 to 101) exhibited comparably robust PLK1 phosphorylation–dependent binding (Fig. 1F), whereas a fragment lacking the predicted γTuNA did not (amino acids 1 to 60; Fig. 1F). Because the amino acids 1–101 and 1–113 fragments both exhibit phosphorylation-regulated γTuC binding, we refer to them interchangeably as PRGB1 (Fig. 1F). In further agreement with the alignment, extending the N-terminal SPD-5 fragment beyond PRGB1 to include the region with homology to γTuNA-In (amino acids 1 to 137 and 1 to 170) strongly suppressed PLK1 phosphorylation–stimulated γTuC binding (Fig. 1, D and E). Two additional N-terminal SPD-5 fragments (1–200 and 1–270), which include the PLK1 phosphorylation sites previously shown to be important for γTuC binding in vitro and recruitment to centrosomes in vivo (T178 and T198) (*38*), also failed to exhibit substantial PLK1 phosphorylation–dependent binding (Fig. 1, E and G). However, consistent with the robust phosphorylation-dependent binding previously observed for the N-terminal half of SPD-5 (amino acids 1 to 473) (*38*), further extension of the N-terminal region to amino acid 345 or 473 restored PLK1 phosphorylation–dependent γTuC binding (Fig. 1, G and H).

The alternating pattern of presence, loss, and recovery of γTuC binding across this series of N-terminal SPD-5 fragments (Fig. 1H) indicates that the PLK1 phosphorylation–controlled mechanisms that convert the SPD-5 N terminus into a γTuC docking site are complex. This pattern motivated us to undertake a detailed investigation of the conversion mechanism, beginning with analysis of γTuC binding by PRGB1.

### Phosphorylation converts the region preceding the γTuNA from inhibitory to activating for γTuC binding

The results above indicated that PRGB1, which consists of the predicted γTuNA preceded by 60 N-terminal amino acids, exhibits robust PLK1 phosphorylation–dependent γTuC binding (Fig. 1, F and H). Relative to PRGB1, fragments of PRGB1 lacking the first 30 or 60 amino acids exhibited elevated γTuC binding in the absence of phosphorylation, with only modest enhancement upon PLK1 phosphorylation (SPD-5 amino acids 59 to 113, Fig. 1F; amino acids 31 to 113, fig. S1A). This result has two implications. First, it suggests that the extreme N terminus of SPD-5 (amino acids 1 to 60) inhibits phosphorylation-independent γTuC binding by the γTuNA and that this inhibition is relieved by PLK1 phosphorylation. Our prior work identified two candidate PLK1 target sites in the first 30 amino acids of SPD-5 that are broadly conserved across metazoans (S5 and S11; fig. S1B) (*38*). Mutating S5 and S11 to alanine reduced the ability of PLK1 to stimulate γTuC binding by PRGB1 (Fig. 1I), suggesting that phosphorylation of these sites contributes to relief of γTuNA inhibition by the amino acids 1–60 region (Fig. 1J). Second, the observation that PLK1-phosphorylated PRGB1 binds γTuCs better than the smaller fragment containing only the γTuNA (Fig. 1F) suggests that phosphorylation of amino acid 1 to 60 not only relieves autoinhibition but also contributes positively to γTuC binding (Fig. 1J). In contrast, fragments containing PRGB1 followed by the region homologous to the γTuNA-In (SPD-5 amino acids 1 to 137 or 1 to 170) could not be activated by phosphorylation (Fig. 1E), indicating that addition of this predicted α-helical region suppresses γTuC binding in a way that renders PRGB1 refractory to PLK1-mediated activation (Fig. 1J).

**Fig. 2.**
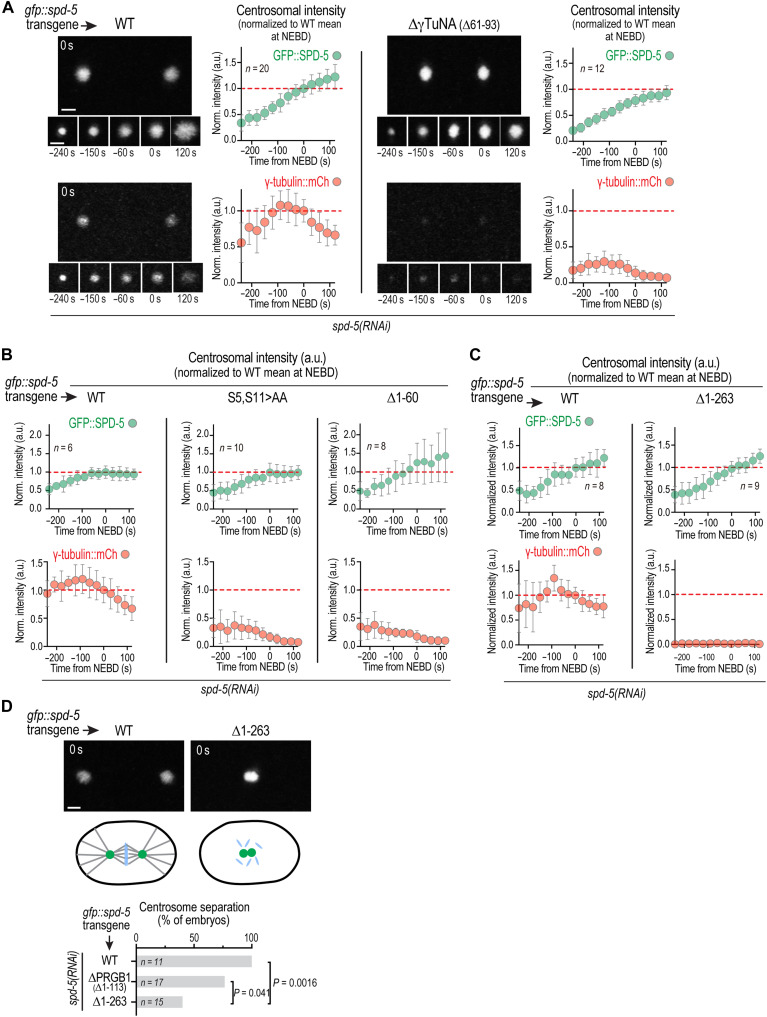
Impact of deletions in the SPD-5 N terminus on γTuC recruitment to centrosomes. (**A** to **C**) Images and quantification (A) or quantification only (B and C) of centrosomal fluorescence intensity over time for γ-tubulin::mCherry and the indicated GFP::SPD-5 variants after depletion of endogenous SPD-5 by RNA interference (RNAi). Centrosomal fluorescence was normalized by dividing by the mean at NEBD for WT GFP::SPD-5. The means for the WT control at NEBD are marked with a red dashed line. Error bars are the standard deviation (SD). *n* is the number of centrosomes analyzed from at least three biological replicates imaged for each condition. (**D**) Top: Images of an embryo expressing WT GFP::SPD-5 with separated centrosomes and one expressing GFP::SPD-5 Δ1–263 that failed to separate its centrosomes. Middle: Schematics illustrating the location of the centrosomes and status of the spindle in the imaged embryos. Bottom: Graph plots the percentage of embryos with separated centrosomes expressing each of the indicated GFP::SPD-5 variants. Endogenous SPD-5 was depleted by RNAi under all conditions; *n* is the number of imaged embryos from at least five biological replicates. Scale bars, 2 μm. *P* values are from one-sided Fisher’s exact test. Note that separate WT controls are shown for (A), (B), and (C) because the data were collected at different times on different microscopes. a.u., arbitrary units.

To determine whether these in vitro observations align with the in vivo requirements for γTuC recruitment to centrosomes, we imaged embryos expressing γ-tubulin::mCherry together with RNA-resistant single-copy transgene-encoded wild-type (WT) or mutant green fluorescent protein (GFP)::SPD-5, following depletion of endogenous SPD-5 (Fig. 2, A to C). Relative to embryos expressing untagged SPD-5—in which γ-tubulin::mCherry levels remain high between nuclear envelope breakdown (NEBD) and anaphase onset before declining (*38*)—embryos expressing GFP-tagged SPD-5 exhibited slightly altered γ-tubulin::mCherry dynamics. Specifically, γ-tubulin::mCherry reached a similar maximal level but began to decline earlier when the transgene-encoded SPD-5 was GFP-tagged versus when it was untagged SPD-5 (fig. S1C). All comparisons were of GFP-tagged SPD-5s, allowing direct comparison of γTuC recruitment.

Using this system, we found that deletion of the core γTuNA homology region (Δ61–93), truncation of the first 60 amino acids (Δ60), or mutation of S5 and S11 to alanine did not impair assembly of the SPD-5 matrix around centrioles during mitotic entry (Fig. 2, A and B). In contrast, all three perturbations caused a similarly strong reduction in γTuC recruitment (Fig. 2, A and B). These results confirm the importance of the γTuNA homology region and the first 60 amino acids of SPD-5 for γTuC recruitment in vivo and support the conclusion from the biochemical analysis that phosphorylation of the 1–60 region, which includes Ser^5^ and Ser^11^, both alleviates γTuNA inhibition and contributes positive affinity to γTuC binding (Fig. 1, F, I, and J).

### Identification of a second region in the SPD-5 N terminus that is sufficient for PLK1-regulated γTuC binding

Given the emphasis on the γTuNA as a central element of the γTuC docking site, we anticipated that γTuC recruitment would be abolished upon deletion of this motif. However, residual γ-tubulin was detected at centrosomes in embryos expressing GFP::SPD-5 lacking the γTuNA (Fig. 2A). In contrast, γ-tubulin fell below the level of detection in embryos expressing a larger N-terminal truncation of SPD-5 (∆1–263), despite normal assembly of the SPD-5 matrix around centrioles (Fig. 2C). Consistent with these observations, centrosomes successfully separated in the majority of embryos expressing SPD-5 lacking PRGB1 (Fig. 2D; ∆1–113), indicating partial microtubule-nucleating capacity, whereas centrosomes failed to separate in the majority of embryos expressing the larger ∆1–263 truncation (Fig. 2D). Together, these results hinted that the SPD-5 N terminus may contain a second PRGB1-independent means of interacting with γTuCs.

Immediately following PRGB1 is the predicted α helix 2 (amino acids 118 to 166; Fig. 1D). Addition of the region containing the γTuNA-In (SPD-5 amino acids 1 to 137), or all of predicted helix 2 (SPD-5 amino acids 1 to 170), to PRGB1 strongly suppressed PLK1 phosphorylation–stimulated γTuC binding (Fig. 1E), whereas further extension of the N terminus to amino acid 345 restored phosphorylation-dependent binding (Fig. 1G). These observations suggest that, in addition to a potential PRGB1-independent γTuC-binding interface, distal elements of the SPD-5 N terminus are required to overcome the potent inhibition of PRGB1 imposed by the γTuNA-In, which otherwise renders it unresponsive to PLK1 phosphoregulation.

**Fig. 3.**
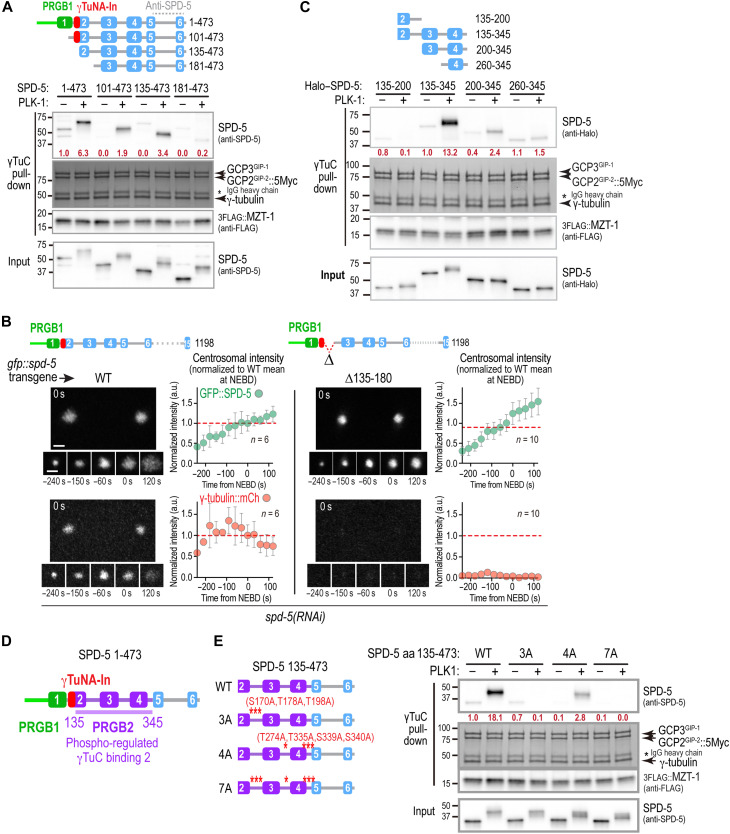
Identification of a second region in the SPD-5 N terminus that is sufficient for PLK1-regulated γTuC binding. (**A** and **C**) Binding assays with γTuC-coated beads and the indicated untagged (A) or Halo-tagged (C) SPD-5 fragments, preincubated with or without PLK1 as indicated. SPD-5 and MZT-1 were analyzed by immunoblotting using the indicated antibodies; γ-tubulin, GCP2^GIP-2^, and GCP3^GIP-1^ were detected by Coomassie staining. Numbers in dark red below the SPD-5 fragment bands indicate band intensity relative to the SPD-5 1–473 (A) or SPD-5 135–345 (C) in the absence of PLK1 phosphorylation. Asterisk indicates the IgG heavy chain of the anti-Myc antibody used for the Myc immunoprecipitation (IP). (**B**) Images and quantification of centrosomal fluorescence intensity over time for γ-tubulin::mCherry and the indicated GFP::SPD-5 variants after depletion of endogenous SPD-5 by RNAi. Centrosomal fluorescence was normalized by dividing by the mean at NEBD for WT GFP::SPD-5. The means for the WT control at NEBD are marked with a red dashed line. Error bars are the SD. *n* is the number of centrosomes imaged from at least three biological replicates for each condition. Scale bars, 2 μm. (**D**) Schematic showing the relative locations in the SPD-5 N terminus of the two independent phosphorylation-regulated γTuC binding elements, PRGB1 and PRGB2, and the intervening inhibitory γTuNA-In element. (**E**) Binding assays with γTuC-coated beads performed as in (A) and (C) for the indicated SPD-5 variants. Numbers in dark red below the SPD-5 fragment bands indicate band intensity relative to SPD-5 135–473 in the absence of PLK1 phosphorylation.

To understand the roles of the region of SPD-5 distal to PRGB1 and the γTuNA-In, we analyzed γTuC binding by a series of N-terminal truncations of SPD-5 amino acids 1 to 473. Fragments lacking PRGB1 (amino acids 101 to 473) or lacking both PRGB1 and the γTuNA-In (amino acids 135 to 473) exhibited robust PLK1 phosphorylation–dependent γTuC binding (Fig. 3A). In contrast, a shorter fragment lacking all of predicted α helix 2 (amino acids 181 to 473) failed to bind γTuCs in the presence or absence of PLK1 (Fig. 3A). These results indicate that amino acids 135 to 180, encompassing the portion of α helix 2 distal to the γTuNA-In, is an essential part of a second, PRGB1-independent γTuC-binding interface (Fig. 3A). In vivo, GFP-tagged SPD-5 lacking amino acids 135 to 180 assembled normally around centrioles but reduced γTuC recruitment to near background levels (Fig. 3B). This result is consistent with the idea that the distal region of the SPD-5 N terminus has dual roles in γTuC binding and in disengaging PRGB1 from the γTuNA-In. Biochemical analysis further revealed that SPD-5 amino acids 135 to 345 exhibited robust PLK1 phosphorylation–dependent γTuC binding, whereas smaller fragments lacking either the N-terminal (amino acids 200 to 345) or C-terminal (amino acids 135 to 200) portions of this region did not (Fig. 3C). Thus, both amino acids 135 to 180, which includes the distal portion of α helix 2, and amino acids 200 to 345, which contains predicted α helices 3 and 4, are required for phosphorylation-dependent γTuC binding. Notably, amino acids 200 to 345 are also required to alleviate inhibition of PRGB1 by the γTuNA-In (Fig. 1, E and G). Collectively, these results define a second region of SPD-5 (amino acids 135 to 345) that is sufficient for PLK1 phosphorylation–dependent γTuC binding and is additionally required to alleviate γTuNA-In–mediated inhibition of PRGB1. We therefore designate this region PRGB2 (Fig. 3D).

PRGB2 contains three PLK1 phosphorylation sites between predicted helices 2 and 3 (S170, T178, and T198), two of which (T178 and T198) were previously shown to be important for γTuC binding in vitro and centrosomal recruitment in vivo (*38*). PRGB2 also contains four additional predicted PLK1 phosphorylation sites within the loops between predicted helices 3 and 4 and 4 and 5 (Fig. 3E and fig. S1B). To assess the contribution of these sites to PRGB2-mediated γTuC binding, we compared WT and alanine-mutated N-terminal fragments lacking PRGB1 (Fig. 3E). This analysis revealed that residues S170, T178, and T198 are important for γTuC binding by PRGB2 and that the four additional C-terminal PLK1 target sites also contribute to binding (Fig. 3E). Although reduced in magnitude, a PLK1 phosphorylation–dependent mobility shift was still observed for the 7A mutant, indicating the presence of additional PLK1 target sites beyond the ones mutated. Together, these results identify PRGB2 as a second PLK1-regulated γTuC binding region within the SPD-5 N terminus.

### PRGB2, but not PRGB1, requires the MZT-1:GCP3-NHD module for binding to γTuCs

We next sought to define the elements of the γTuC that are required for binding to PLK1-phosphorylated PRGB1 and PRGB2. The reconstituted *C. elegans* γTuC used in our binding assays contains γ-tubulin, GCP2^GIP-2^, GCP3^GIP-1^, and MZT-1, and the *C. elegans* homolog of Mozart 1 (MZT-1) (*21*, *38*, *66*, *67*). MZT-1 is a small, widely conserved microprotein composed of three α helices that is required for γTuCs to interact with tethering proteins at MTOCs across eukaryotes (*23*, *50*). MZT-1 intercalates with the NHDs of the γTuSC subunit GCP3 as well as with other γTuRC-specific GCPs (*23*, *30*, *43*, *45*, *51*–*59*). A second vertebrate-specific Mozart protein, MZT-2, similarly intercalates with the NHD of GCP2; in humans, the CDK5RAP2 γTuNA forms a parallel coiled-coil that interacts with the MZT-2:GCP2-NHD module (*30*, *45*, *47*, *48*, *58*). In *C. elegans*, the single Mozart-family protein, MZT-1, is required for γTuCs to dock onto the PLK1-phosphorylated SPD-5 matrix at centrosomes and for the SPD-5 N terminus (amino acids 1 to 473) to bind γTuCs following PLK1 phosphorylation (*38*, *67*). We therefore tested whether the MZT-1–containing module is required for phosphorylated PRGB1 and PRGB2 to interact with γTuCs.

**Fig. 4.**
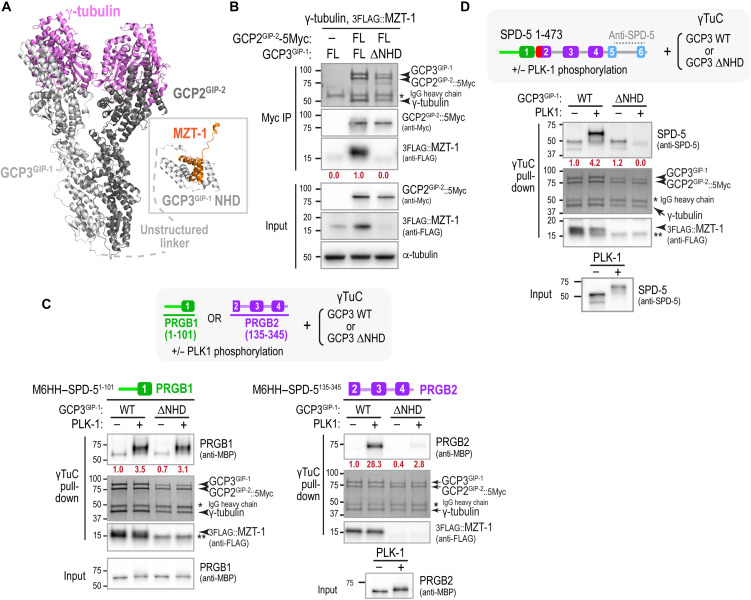
PRGB2, but not PRGB1, requires the MZT-1 module for binding to γTuCs. (**A**) AlphaFold 3 model of the *C. elegans* γTuC. The MZT-1 module comprised MZT-1, and the GCP3^GIP-1^ NHD is separated from the core tetrameric complex by an unstructured linker and is not positioned relative to the core complex. See also fig. S2A. (**B**) Analysis of the composition of the γTuC when either full-length (FL) GCP3^GIP-1^ or GCP3^GIP-1^ with its NHD deleted (ΔNHD) was expressed. A condition in which GCP2^GIP-2^ with the Myc tag used for the immunoisolation omitted is shown as a control. MZT-1 and GCP2^GIP-2^ were detected by Western blotting; γ-tubulin, GCP2^GIP-2^, and GCP3^GIP-1^ were detected by Coomassie staining. Numbers in dark red below the MZT-1 bands indicate band intensity relative to MZT-1 levels when FL GCP3^GIP1^ was expressed. Asterisk indicates the IgG heavy chain of the anti-Myc antibody used for the Myc IP. Note that MZT-1 is unstable in the absence of the GCP3^GIP-1^ NHD, so its levels are reduced in the input as well as in the immunoprecipitated complex. (**C** and **D**) Pull-down experiments with beads coated with γTuCs assembled with WT or ΔNHD GCP3^GIP-1^. Beads were incubated with the indicated SPD-5 fragments after preincubation with or without PLK1 as indicated. SPD-5 and MZT-1 were analyzed by immunoblotting using the indicated antibodies; γ-tubulin, GCP2^GIP-2^, and GCP3^GIP-1^ were detected by Coomassie staining. Numbers in dark red below the SPD-5 fragment bands indicate band intensity relative to PRGB1 [(C), left], PRGB2 [(C), right] or SPD-5 1–473 (D) in the absence of PLK1 phosphorylation. The single asterisk indicates the IgG heavy chain of the anti-Myc antibody used for the Myc IP. The double asterisk marks the location of a nonspecific band.

An AlphaFold 3 structural model of the reconstituted *C. elegans* γTuC predicted a canonical heterotetrameric small γTuC (Fig. 4A). In this model, GCP3^GIP-1^, but not GCP2^GIP-2^, contains an NHD predicted to form an intercalated module with MZT-1, which we term the MZT-1:GCP3-NHD module (Fig. 4A). Consistent with this prediction, expression of GCP3^GIP-1^ lacking its NHD produced γTuCs that failed to incorporate MZT-1 (Fig. 4B). The MZT-1:GCP3-NHD module is connected to the rest of GCP3^GIP-1^ by an unstructured region and is not predicted to adopt a defined position relative to the rest of the γTuC (Fig. 4A and fig. S2A). Moreover, modeling assemblies containing two or more γTuCs did not yield high-confidence predictions for the relative placement of the MZT-1:GCP3-NHD module with respect to other subunits.

We next compared binding of phosphorylated PRGB1 and PRGB2 to γTuCs that either contained or lacked the MZT-1:GCP3-NHD module. Notably, phosphorylated PRGB2 required the MZT-1:GCP3-NHD module for γTuC binding, whereas PRGB1 did not (Fig. 4C and fig. S2B). These results indicate that phosphorylated PRGB1 and PRGB2 engage γTuCs through distinct interfaces and can bind independently of one another when tested in isolation.

In addition to mediating γTuC binding, PRGB2 is required to relieve inhibition of PRGB1 by the γTuNA-In, which otherwise renders PRGB1 refractory to PLK1-dependent activation. Consistent with this dual role, binding of the full SPD-5 N terminus (amino acids 1 to 473) to γTuCs depended on the MZT-1:GCP3-NHD module that supports PRGB2 engagement (Fig. 4D). Together, these data suggest that when PRGB2 cannot interface with the MZT-1:GCP3-NHD module, PRGB1 fails to form its independent γTuC-binding interface_,_ potentially because PRGB1 cannot be disengaged from γTuNA-In–mediated inhibition.

### PRGB2 dimerizes the SPD-5 N terminus and undergoes a conformational change upon PLK1 phosphorylation

**Fig. 5.**
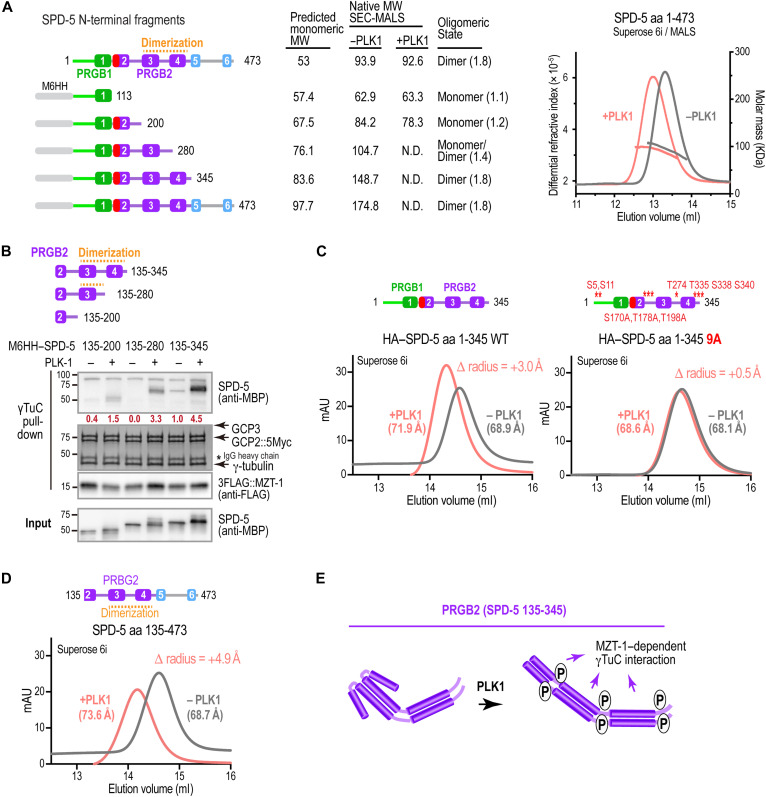
The SPD-5 N terminus is dimerized by PRGB2 and undergoes a conformational change upon PLK1 phosphorylation. (**A**) Left: Schematics of untagged and M6HH-tagged SPD-5 variants that were analyzed by SEC-MALS. Middle: Table summarizing SEC-MALS results, listing predicted monomeric molecular weight, native molecular weight measured by SEC-MALS with or without PLK1 phosphorylation, and inferred oligomerization state [native/predicted molecular weight (MW) ratio in parentheses]. N.D., not determined. Right: Example SEC-MALS data for untagged SPD-5 amino acids 1 to 473 before and after PLK1 phosphorylation. Although phosphorylated and unphosphorylated dimers have the same native molecular weight, phosphorylated dimers elute earlier, indicating an increased hydrodynamic radius. (**B**) Binding assays with γTuC-coated beads and the indicated M6HH-tagged SPD-5 fragments, preincubated with or without PLK1 as indicated. SPD-5 and MZT-1 were analyzed by immunoblotting using the indicated antibodies; γ-tubulin, GCP2^GIP-2^, and GCP3^GIP-1^ were detected by Coomassie staining. Numbers in dark red below the SPD-5 fragment bands indicate band intensity relative to SPD-5 135–345 in the absence of PLK1 phosphorylation. Asterisk indicates the IgG heavy chain of the anti-Myc antibody used for the Myc IP. (**C**) SEC analysis of HA-tagged SPD-5 amino acids 1 to 345 (left) or the same fragment with nine predicted PLK1 sites mutated to alanines (right). Hydrodynamic radii were calculated based on column calibration with standard proteins and are shown in parentheses; the phosphorylation-induced change in hydrodynamic radius is indicated in the upper right of each graph. (**D**) SEC analysis of SPD-5 amino acids 135 to 473, which lacks PRGB1 and γTuNA-In, following preincubation with or without PLK1. Hydrodynamic radii are shown as in (C). (**E**) Speculative model illustrating how PLK1 phosphorylation promotes a conformational change in PRGB2 that both increases hydrodynamic radius and positively contributes to γTuC binding affinity. mAU, milli-absorbance units.

We next asked how phosphorylation of PRGB2 promotes γTuC interaction. PRGB2 comprised predicted coiled-coils, raising the possibility that coiled-coil–mediated dimerization contributes to γTuC binding. To test this, we analyzed purified SPD-5 fragments by size exclusion chromatography coupled to multiangle light scattering (SEC-MALS) (Fig. 5A and fig. S3, A and B). The largest SPD-5 N-terminal fragment (amino acids 1 to 473) exhibited a native molecular weight consistent with a dimer, both when untagged and when fused to a monomeric maltose-binding protein (MBP) tag, as did an MBP-tagged version of the amino acids 1–345 fragment (Fig. 5A and fig. S3A). Phosphorylation by PLK1 did not alter the native molecular weight of the amino acids 1–473 fragment (Fig. 5A and fig. S3A), indicating that the SPD-5 N terminus is dimeric independent of phosphorylation status. In contrast, PRGB1 alone (SPD-5 amino acids 1 to 113) and the longer amino acids 1–200 fragment had native molecular weights consistent with monomers, regardless of PLK1 phosphorylation status (Fig. 5A and fig. S3B). The amino acids 1–280 fragment, which contains predicted helix 3 but not helix 4, exhibited an intermediate molecular weight consistent with a monomer-dimer equilibrium (Fig. 5A and fig. S3A). Together, the SEC-MALS analysis indicates that dimerization of the SPD-5 N terminus depends on α helices 3 and 4 (Fig. 5A). To relate dimerization to γTuC binding, we compared binding of an MBP fusion with PRGB2 (amino acids 135 to 345), which binds robustly, to that of constructs lacking helix 4 (amino acids 135 to 280) or lacking helices 3 and 4 (amino acids 135 to 200). Whereas weak phosphorylation-stimulated binding was observed when only helix 4 was removed, removal of both helices nearly eliminated binding. Thus, PRGB2-mediated γTuC binding correlates with dimerization mediated by helices 3 and 4 (Fig. 5B).

Although phosphorylation did not change the native molecular weight of the amino acids 1–473 dimer, the phosphorylated form eluted substantially earlier than the unphosphorylated form on the size exclusion column (Fig. 5A). Because SEC separates proteins based on hydrodynamic radius, this earlier elution suggests that phosphorylation increases the hydrodynamic radius of the dimer, consistent with a more extended conformation. This effect mapped to PRGB2-containing fragments: A phosphorylation-dependent elution shift was observed for fragments containing PRGB2 (SPD-5 amino acids 1 to 345 and 135 to 473; Fig. 5, C to E) but not fragments containing only PRGB1 or PRGB1 and the γTuNA-In (SPD-5 amino acids 1 to 113 and 1 to 200; fig. S3B). To confirm that the elution shift was due to phosphorylation, we mutated nine predicted, high-confidence PLK1 sites in SPD-5 (amino acids 1 to 345), which includes PRGB1, the γTuNA-In, and PRGB2. The nine sites included three sets implicated in PLK1-stimulated γTuC binding in vitro [S5 and S11, Fig. 1I; S170, T178, and T198, Fig. 3E and (*38*); and T274, T335, S339, and S340, Fig. 3E]; the first two sets are also important for the centrosomal recruitment of γ-tubulin in vivo (Fig. 2B) (*38*). The PLK1-dependent elution shift was largely abolished in the 9A mutant (Fig. 5C), whereas mutation of either the first five or the last four reduced, but did not eliminate, the shift (fig. S3C).

Together, these results indicate that the SPD-5 N terminus, which contains two separable γTuC-binding interfaces, is a constitutive dimer that undergoes a PLK1 phosphorylation–stimulated conformational change as the result of distributed phosphorylation across the N terminus. Notably, a substantial elution shift persisted following mutation of the five N-terminal sites that are critical for γTuC binding and centrosomal recruitment (fig. S3C; 5A mutant), suggesting that the conformational change may be necessary but not sufficient for γTuC binding. In addition to promoting a more extended conformation, phosphorylation of these residues likely contributes directly to γTuC-binding affinity. Collectively, these data support a model in which PLK1 phosphorylation remodels the dimeric SPD-5 N terminus and enhances its interaction with γTuCs.

### PLK1 phosphorylation disrupts a potentially inhibitory interaction between PRGB1 and PRGB2

**Fig. 6.**
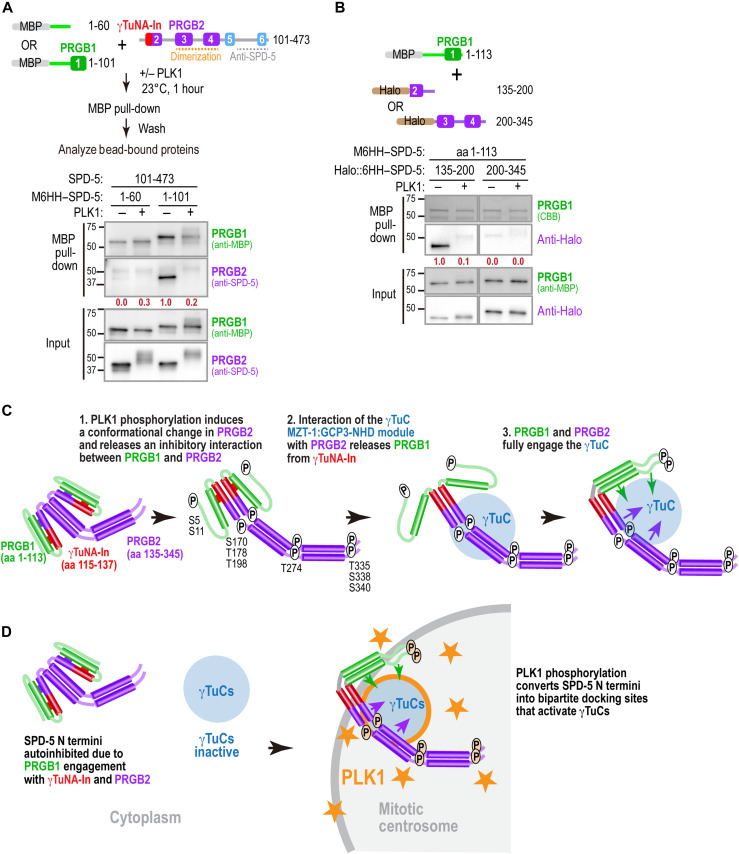
PLK1 phosphorylation disrupts an interaction between PRGB1 and PRGB2. (**A**) Schematic (top) and results (bottom) of a binding experiment in which binding of M6HH-tagged PRGB1 or SPD-5 amino acids 1 to 60 (lacking the γTuNA) to untagged SPD-5 amino acids 101 to 473 containing the γTuNA-In and PRGB2 was assessed. Mixtures were incubated with or without PLK1, and bead-bound proteins were detected by immunoblotting after an MBP-pull-down. Numbers in dark red indicate PRGB2 band intensity relative to the SPD-5 1–101 pull-down without PLK1 phosphorylation. (**B**) Schematic (top) and results (bottom) of an experiment in which binding of M6HH-tagged SPD-5 PRGB1 to the indicated Halo-tagged regions of PRGB2 was analyzed after performing a pull-down in the presence or absence of PLK1 as in (A). Bead-bound proteins were detected by immunoblotting using the indicated antibodies or Coomassie staining. Numbers in dark red below the Halo-tagged fragments are band intensity of the halo-tagged fragment relative to amount of SPD-5 135–200 pulled down in the absence of PLK1 phosphorylation. (**C**) Model for phosphorylation-dependent conversion of the SPD-5 N terminus into a γTuC docking site. PLK1 phosphorylation stimulates a conformational change that extends the dimeric PRGB2 region and releases an inhibitory interaction between PRGB1 and α helix 2 within PRGB2. Interaction of PRGB2 with the MZT-1 module of the γTuC releases PRGB1 from inhibition by the γTuNA-In, enabling PRGB1 to engage the γTuC. Phosphorylation may also directly enhance PRGB1 and PRGB2 affinity for the γTuC. (**D**) Model for the functional importance of PLK1 regulation of γTuC docking. The multistep process required to convert the SPD-5 N terminus into a γTuC docking site ensures that it is fully inhibited in the cytoplasm and binds γTuCs only on the centrosomal scaffold where PLK1 activity is concentrated. Tight control facilitates precise patterning of microtubule nucleation to enable rapid, robust spindle assembly.

The predicted α helix 2 that follows PRGB1 contains both the γTuNA-In and a region (amino acids 135 to 180) required for γTuC-binding by PRGB2 (Fig. 3D). These features suggest a model in which α helix 2 interacts with the γTuNA to mutually inhibit γTuC binding by both PRGB1 and PRGB2 in the unphosphorylated state. To test this idea, we performed binding assays between PRGB1 and SPD-5 fragments containing part or all of PRGB2. PRGB1 (SPD-5 amino acids 1 to 101), but not SPD-5 amino acids 1 to 60—which lacks the γTuNA—bound to SPD-5 amino acids 101 to 473, a fragment that contains both the γTuNA-In and PRGB2 (Fig. 6A). In addition, PRGB1 (amino acids 1 to 113) bound to SPD-5 amino acids 135 to 200, corresponding to the portion of α helix 2 distal to the γTuNA-In that is required for PRGB2-mediated γTuC binding, but did not bind to SPD-5 amino acids 200 to 345 (Fig. 6B). Notably, the interaction between PRGB1 and SPD-5 amino acids 135 to 200 was disrupted by PLK1 phosphorylation. Collectively, these results suggest that PLK1 phosphorylation releases an inhibitory interaction between PRGB1 and PRGB2 (Fig. 6C). This release alone is not sufficient to allow γTuC binding by N-terminal fragments containing α helices 1 and 2 because PRGB1 is also inhibited by the γTuNA-In. As shown above, relief of γTuNA-In–mediated inhibition requires engagement of PRGB2 with the MZT-1:GCP3-NHD module of the γTuC, thereby coupling phosphorylation-dependent conformational changes in SPD-5 to γTuC recruitment.

## DISCUSSION

During mitotic entry in metazoans, centrosomes generate a localized burst of microtubule nucleation to catalyze assembly of a bipolar spindle (*1*–*4*, *7*–*9*). This spatiotemporal control occurs because centrosomally localized PLK1 kinase drives coordinated expansion of the PCM matrix and the generation of mitotic γTuC docking sites on the expanding matrix (*10*–*16*, *38*, *65*), yet how PLK1 phosphorylation remodels matrix molecules to generate productive γTuC docking sites has been unclear. Here, we investigate how PLK1 phosphorylation converts the N terminus of the *C. elegans* CDK5RAP2-family protein SPD-5 into a γTuC docking site. We show that the SPD-5 N terminus contains two separable regions capable of phosphorylation-regulated γTuC binding, which we term PRGB1 and PRGB2, that flank an inhibitory sequence with homology to the γTuNA-In previously described in human CDK5RAP2 (*49*). PRGB1 contains a γTuNA-like region and PRGB2 forms a distinct interface that depends on the MZT-1:GCP3-NHD module of the γTuC. Based on our data, we propose a sequential conversion model (Fig. 6D) in which PLK1 phosphorylation (i) triggers a conformational change that extends the dimeric coiled-coil containing PRGB2, (ii) disrupts an inhibitory interaction between PRGB1 and PRGB2, and (iii) enables PRGB2 to engage the γTuC in an MZT-1:GCP3-NHD module–dependent fashion. Engagement of PRGB2 then overcomes γTuNA-In–mediated inhibition to allow PRGB1 to bind the γTuC. This multistep conversion process likely prevents PRGB1 and PRGB2 from productively engaging γTuCs in the cytoplasm, thereby concentrating γTuC-mediated microtubule nucleation within the high PLK1 activity environment of the centrosomal scaffold to facilitate rapid, robust spindle assembly. An additional key point is that PLK1 phosphorylation contributes not only by relieving autoinhibition, but also by increasing binding affinity for the γTuC and remodeling the conformation of the SPD-5 N terminus. Recent structural work characterizing how the phospho-regulated multimerization (PReM) region in *Drosophila* Cnn is regulated during mitotic entry suggests that PLK1 may also simultaneously relieve autoinhibition and promote new interactions to enable PCM matrix expansion (*68*).

CDK5RAP2-family proteins contain a conserved CM1 motif (*40*, *41*) that includes an ~30–amino acid γTuNA region capable of binding and activating γTuCs (*42*–*48*), followed by an autoinhibitory region (γTuNA-In) that prevents the γTuNA from interacting with γTuCs (*49*). Work on the *Drosophila* CDK5RAP2-family protein Cnn has suggested that the CM1 region is also preceded by a phosphorylation-regulated autoinhibitory domain (*39*). Our analysis shows that the γTuNA-like region of *C. elegans* SPD-5 (amino acids 59 to 101) is similarly flanked by inhibitory regions on both sides. Inhibition by its N-terminally flanking sequence (amino acids 1 to 60) is released by PLK1 phosphorylation, whereas inhibition of the γTuNA by the C-terminal γTuNA-In is not relieved by phosphorylation alone and is instead overcome only after the distally located phosphorylated PRGB2 region engages the γTuC. Given the broad conservation of the CM1 region (*23*, *40*, *49*, *69*) and the presence of functionally similar γTuNA-In–like regions in human CDK5RAP2 (*49*) and *C. elegans* SPD-5 (Fig. 1, D and E), it is likely that the bipartite organization of a γTuC-binding γTuNA region followed by an inhibitory γTuNA-In region will be conserved in CDK5RAP2-family proteins. The mechanisms by which this inhibition is relieved, and the extent to which overcoming this inhibition will require additional γTuC-engaging elements, will be interesting to investigate in different systems.

Our work identified a critical interaction between phosphorylated PRGB2 in SPD-5 and the γTuC. Deletion of a small predicted coiled-coil at the beginning of PRGB2 abolished centrosomal γTuC recruitment (Fig. 3B). Notably, PRGB2 engagement requires the MZT-1:GCP3-NHD module of the γTuC, a conserved interface that plays a central role in tethering γTuCs to MTOCs across eukaryotes (*23*, *30*, *43*, *45*, *51*–*59*). Recent structural work in vertebrates has shown that four of its five MZT-1:GCP3-NHD modules release from the γTuRC spokes and associate to form a pinwheel-like structure around a central tetrameric four-helix bundle “spoke” derived from a region of the γTuRC adapter protein neural precursor cell expressed, developmentally down-regulated 1 (NEDD1) (*45*, *56*). The region of NEDD1 that forms this bundle consists of a pair of linked α helices that form coiled-coils, two of which appear to come together and form a four-helix bundle (*45*, *56*). PRGB2 consists of three linked α helices, two of which appear to form coiled-coils that dimerize the region (Fig. 5A), raising the possibility that the MZT-1:GCP3-NHD module could interact with it in a similar manner. Further work will be needed to understand the conformational change that PRGB2 undergoes upon PLK1 phosphorylation that enables it to engage with the γTuC. It will also be interesting to compare this interaction to that of other MZT-1:GCP3-NHD–dependent interactions that tether γTuCs to MTOCs.

In addition to the PRGB2 motif, which mediates an MZT-1:GCP3-NHD–dependent interaction with the γTuC, SPD-5 has a divergent γTuNA-containing PRGB1 motif that is also required for robust recruitment of γTuCs to the SPD-5 matrix. Our data suggest that PRGB1 is monomeric in vitro and may bind γTuCs without dimerizing. In vertebrates, the γTuNA dimerizes to form a coiled-coil, and a vertebrate-specific Mozart protein (MZT-2) intercalates with the GCP2 NHD to enable an interaction with the γTuNA coiled-coil that promotes closure of the γTuRC into an activated conformation (*30*, *45*–*48*, *58*). Outside of vertebrates—where MZT-2 is absent—how γTuNA-like motifs engage γTuCs and whether they contribute to γTuC activation is less clear. Structural work on dimers of the budding yeast protein Spc110 suggests that its two CM1 domains interface with the γTuSC in distinct ways. A single CM1 domain helix from one protomer was observed to span the inter-γTuSC interface, while the CM1 domain from the second protomer took a different path that was not well resolved (*70*). More structural work will be needed to understand the interactions between γTuNA-like regions and γTuCs outside of vertebrates and the relationship between these interfaces and the degree of conservation of the KENF motif.

Spc110 is also an example of a CM1 domain-containing protein that, like SPD-5, uses two adjacent motifs to bind γTuSCs in a phosphorylation-dependent fashion (*69*). Spc110 belongs to the pericentrin (PCNT) family of γTuC docking proteins, which are defined by the presence of an SPM-CM1 pair of γTuC-interacting motifs in their N termini. By contrast, CDK5RAP2 family proteins, including Cnn and SPD-5, have a CM1 region without an SPM motif (*69*). In Spc110, the SPM and CM1 motifs are separated by a set of activating regulatory phosphorylation sites. When phosphorylated, the Spc110 N terminus uses its SPM and CM1 motifs to promote the lateral interaction of γTuSCs to form large spiral assemblies that promote microtubule nucleation (*69*). In vivo work has suggested that the SPD-5 based PCM matrix in *C. elegans* can recruit γTuCs containing the small complex components (γ-tubulin, GCP2, GCP3, and MZT-1) but lacking the potential γTuRC components GTAP-1 and GTAP-2 to support normal levels of centrosomal microtubule nucleation (*31*). Structural analysis that incorporates PLK1 phosphorylation will be needed to test if SPD-5 can use its PRGB1 and PRGB2 motifs to promote the oligomerization of *C. elegans* γTuSCs to form larger complexes that nucleate microtubules at centrosomes.

## MATERIALS AND METHODS

### *C. elegans* strains and transgene generation

*C. elegans* strains (listed in table S1) were maintained at 16°C. Single-copy transgenes were generated using the MosSCI transposon-based method (*71*) to insert them at defined chromosomal sites. Transgenes were cloned into pCFJ151, which contains the Cb-unc-119 selection marker and appropriate homology arms. SPD-5 mutant transgenes were generated as described previously (*38*). Because the endogenous *spd-5* promoter contains a highly repetitive sequence that reduces MosSCI efficiency, *spd-5* transgenes were instead driven by the *spd-2* promoter, comprising 3043 base pairs (bp) upstream of the start codon. The SPD-5 coding region was followed by 577 bp downstream of the stop codon, and a 577-bp internal segment was re-encoded to confer resistance to a double-stranded RNA (dsRNA) targeting this region without altering protein coding information. Injection mixes contained the pCFJ151-derived repair plasmid (50 to 100 ng/μl), transposase plasmid pCFJ601 (Peft-3::Mos1 transposase, 50 ng/μl), and four plasmids for negative selection against chromosomal arrays: pMA122 (Phsp-16.41::peel-1, 10 ng/μl), pCFJ90 (Pmyo-2::mCherry, 2.5 ng/μl), pCFJ104 (Pmyo-3::mCherry, 5 ng/μl), and pGH8 (Prab-3::mCherry, 10 ng/μl). Mixes were injected into strains EG6429 or EG6699 to target the ttTi5605 site on Chr II. After 1 week, the progeny of injected worms were heat-shocked at 34°C for 3 hours to induce the expression of PEEL-1 to kill worms containing extra chromosomal arrays. Moving worms lacking fluorescent markers were identified as candidates, and polymerase chain reaction (PCR) across both integration junctions was used to confirm transgene integration in their progeny.

### RNA interference

Single-stranded RNAs (ssRNAs) were synthesized in 50 μl of T3 and T7 transcription reactions (MEGAscript, Invitrogen) using gel-purified DNA templates generated by PCR from N2 genomic DNA with primers containing T3 or T7 promoter sequences (table S2). Transcription products were purified using the MEGAclear kit (Invitrogen). Equal volumes (50 μl) of T3- and T7-generated ssRNA were combined with 50 μl of 3× soaking buffer (32.7 mM Na_2_HPO_4_, 16.5 mM KH_2_PO_4_, 6.3 mM NaCl, and 14.1 mM NH_4_Cl) and annealed by heating at 68°C for 10 min, followed by 37°C for 30 min to form dsRNA. dsRNAs were injected at a concentration of ≥1.3 μg/μl. For live imaging of early embryos after RNA interference (RNAi), L4 hermaphrodites were injected with dsRNAs and incubated at 16°C for 48 hours before dissection to isolate embryos for imaging.

### Antibodies

A previously described antibody against SPD-5 (392 to 550 amino acids) (*72*) was used at 1 μg/ml for immunoblotting. The following commercially available antibodies were used at the indicated dilutions: anti-α-tubulin (DM1A; Sigma-Aldrich; 1:5000), anti-FLAG (F1804; Sigma-Aldrich; 1:1000), anti-Myc (9E10; M4439; Sigma-Aldrich; 1:5000), anti-MBP (ab9084; Abcam; 1:1000), anti-MBP (E8032S; NEB; 1:5000), and anti-Halo (G9211; Promega; 1:1000). Secondary antibodies were obtained from Jackson ImmunoResearch and GE Healthcare.

### Live imaging

Embryos for live imaging experiments were obtained by dissecting gravid adult hermaphrodites in M9 buffer (42 mM Na_2_HPO_4_, 22 mM KH_2_PO_4_, 86 mM NaCl, and 1 mM MgSO_4_). One-cell embryos were transferred with a mouth pipette onto a 2% agarose pad and overlaid with a 22 mm–by–22 mm coverslip. Imaging was performed in a temperature-controlled room at 20°C using spinning-disk or laser-scanning confocal microscopy. Spinning-disk imaging was conducted on either (i) a Zeiss Axio Observer.Z1 inverted microscope equipped with a CSU-X1 (Yokogawa), 63×/1.4 NA Plan-Apochromat objective, and a Photometrics QuantEM: 512SC camera (Fig. 2B); or (ii) an inverted Nikon Eclipse Ti2 microscope equipped with a CSU-X1 (Yokogawa), a 60×/1.4 NA Plan-Apochromat objective (Nikon), and an iXon Life EMCCD camera (iXON-L-888; Andor) (Fig. 2D). Embryos shown in Figs. 2 (A and C) and 3B were imaged using a Zeiss LSM880 confocal microscope equipped with Airyscan and a 63×/1.40 Oil Plan-Apochromat WD0.19 lens (Zeiss) in a 20° to 22°C room. Images were processed with ZEN 2.3 SP1 Black software (Zeiss). Images of centrosomal fluorescence were acquired every 30 s by collecting 11 *z*-planes at 1.5-μm intervals without binning. Imaging was initiated in one-cell embryos between centrosome separation and pronuclear meeting and was terminated after initiation of cytokinesis.

### Image analysis

All images were processed and analyzed using ImageJ (National Institutes of Health). Centrosomal fluorescence was quantified from maximum intensity projections of entire *z*-stacks. To quantify centrosomal fluorescence, a fixed size box was drawn around the centrosome at each time point (smallest box that could enclose the centrosomal signal at their largest point in the image sequence; box size varied depending on marker and imaging conditions), along with a box one pixel larger on each side in both dimensions. The per-pixel background was calculated as [(integrated intensity in the larger box − integrated intensity in the smaller box)/(area of larger box − area of smaller box)]. The centrosomal signal was calculated as the mean intensity in the smaller box minus the per-pixel background. To quantify immunoblot band intensity, a fixed size box was drawn around each band (the smallest box that could enclose the strongest band signal in the blot; box size varied depending on the blot and imaging conditions), along with a box one pixel larger above and below. The per-pixel background was calculated as [(integrated intensity in the larger box − integrated intensity in the smaller box)/(area of larger box − area of smaller box)]. The band intensity was calculated as the mean intensity in the smaller box minus the per-pixel background. For γTuC pull-down assays, the SPD-5 band intensity was normalized by dividing by the corresponding band intensity in the input samples.

### Protein expression and purification

Glutathione S-transferase (GST)-, MBP-, or Halo-tagged fragments of the SPD-5 N terminus were expressed in BL21(DE3) pLysS *Escherichia coli* from DNA constructs cloned into a pGEX-6P-1, pMAL-6His-TEV, or pHalo-6His-TEV vectors, respectively. The pMH-Halo tag plasmid was a gift from M. Huen (Addgene plasmid no. 154144; http://n2t.net/addgene:154144). When bacterial cultures reached an OD_600_ (optical density at 600 nm) of 0.6, protein expression was induced for 16 to 18 hours at 15°C by adding 0.3 mM isopropyl-β-d-thiogalactopyranoside. Cells were washed once with cold phosphate-buffered saline (PBS) and flash-frozen in liquid nitrogen. For GST-tagged proteins, pelleted cells were resuspended in lysis buffer (PBS containing 250 mM NaCl, 10 mM EGTA, 10 mM EDTA, 0.1% Tween, 200 μg/ml lysozyme, 2 mM benzamidine, and EDTA-free protease inhibitor cocktail; Roche). For MBP-6His– or Halo-6His–tagged proteins, cells were resuspended in the same buffer supplemented with 10 mM imidazole. Cell lysis was performed by sonication, and lysates were clarified by centrifugation at 40,000 rpm for 20 min at 4°C in a 45 Ti rotor (Beckman). Cleared cell lysates were incubated with 500 μl of glutathione agarose (Cytiva) or Ni-NTA agarose beads (QIAGEN) for 2 hours at 4°C. The resin was washed twice with 30 ml of wash buffer (PBS containing 250 mM NaCl, 1 mM β-mercaptoethanol, and 2 mM benzamidine) for GST-tagged proteins or with the same wash buffer supplemented with 20 mM imidazole for MBP-6His– or Halo-6His–tagged proteins, followed by incubation with 10 ml of washing buffer containing 5 mM adenosine 5′-triphosphate (ATP) for 10 min at 4°C to reduce nonspecific interactions with heatshock proteins. The resin was then washed three additional times with wash buffer. For GST-tagged proteins, the resin was incubated overnight at 4°C with PreScission protease (Eton Bioscience) or HRV-3C protease (ACRO Biosystems) in elution buffer [20 mM tris-Cl (pH 8.0), 150 mM NaCl, and 1 mM dithiothreitol (DTT)] to elute the untagged SPD-5 N-terminal fragments by cleavage from the GST-tag. The supernatant was collected the next day and stored at 4°C. For MBP-6His– or Halo-6His–tagged proteins, the resin was transferred to a poly-prep chromatography column (Bio-Rad), eluted with buffer containing 250 mM imidazole, and then stored at 4°C. The eluted 6His-tagged proteins were concentrated using 10 kDa Molecular Weight Cut-Off (MWCO) Amicon Ultra concentrators (Millipore) and dialyzed into 20 mM tris-HCl (pH 7.5), 50 mM NaCl, 2 mM MgCl_2_, and 1 mM DTT. C-terminally 6His-tagged PLK1 T194D, purified from baculovirus infected Sf9 cells using the FlexiBAC system (*73*) using previously described protocols (*74*, *75*), was a gift from J. Woodruff (UT Southwestern).

### Purification of reconstituted *C. elegans* γTuCs

For reconstitution of the *C. elegans* γTuC, Myc-tagged GCP2^GIP-2^, FLAG-tagged MZT-1, GCP3^GIP-1^, and γ-tubulin^TBG-1^ were cloned into cytomegalovirus (CMV) promoter-driven human expression vectors (table S3) and cotransfected into FreeStyle 293F cells (Thermo Fisher Scientific). The coding sequences for GCP2^GIP-2^ and γ-tubulin^TBG-1^ were amplified by PCR from an N2 cDNA library, whereas codon-optimized GCP3^GIP-1^ and MZT-1 sequences were synthesized (GENEWIZ). Empty 5Myc plasmid (CS2P; Addgene no. 17095) or 3FLAG plasmid (p3XFLAG-CMVTM-7.1; Sigma-Aldrich no. E7533) served as negative controls. Cell transfection was performed using FreeStyle MAX Reagent and OptiPRO SFM according to the manufacturer’s instructions (Thermo Fisher Scientific). A total of 12.5 μg of plasmid DNA was transfected into 10 ml of cells at 1 × 10^6^ cells/ml. After 43 to 38 hours, cells were harvested, washed with PBS, and resuspended in lysis buffer [20 mM tris-HCl (pH 7.5), 50 mM NaCl, 0.5% Triton X-100, 5 mM EGTA, 1 mM DTT, 2 mM MgCl_2_, and EDTA-free protease inhibitor cocktail; Roche]. Lysis was performed in an ice-cold sonicating water bath for 5 min, followed by centrifugation at 15,000*g* for 15 min at 4°C. Cleared lysates were incubated with Pierce Anti-c-Myc magnetic beads (Thermo Fisher Scientific) for 2 hours at 4°C. Beads were washed five times with lysis buffer and either used directly for pull-down assay or resuspended in SDS sample buffer. For immunoblotting, equal volumes of samples were run on Mini-PROTEAN gels (Bio-Rad) and transferred to polyvinylidene difluoride membranes using a TransBlot Turbo system (Bio-Rad). Blocking and antibody incubations were performed in tris-buffered saline containing 0.1% Tween 20 (TBS-T) plus 5% nonfat dry milk or in TBS-T plus 5% bovine serum albumin. Membranes were incubated with primary antibodies overnight at 4°C and with the secondary antibodies for 1 hour at room temperature. Signals were developed using SuperSignal West Femto Substrate (Thermo Fisher Scientific) and detected using a ChemiDoc XRS+ system (Bio-Rad). For Coomassie staining to detect *C. elegans* γTuC, equal volumes of samples were run on Mini-PROTEAN gels (Bio-Rad) and stained with SimplyBlue Safe Stain (Thermo Fisher Scientific).

### Kinase and pull-down assays

For the γTuC pull-down assays (Figs. 1 and 3 to 5), each purified SPD-5 fragment (final concentration of 2.0 μM) was incubated with constitutively active PLK1(T194D) (final concentration of 500 nM) in kinase buffer [20 mM tris-Cl (pH 7.5), 50 mM NaCl, 10 mM MgCl_2_, 0.2 mM ATP, and 1 mM DTT] for 1 hour at 23°C. Following phosphorylation, the reaction mixtures were combined with γTuCs immobilized on Myc beads in lysis buffer and incubated for 2 hours at 4°C. The final concentration of the SPD-5 fragments was 75 nM. Beads were washed five times with lysis buffer and resuspended in sample buffer prior to analysis on SDS–polyacrylamide gel electrophoresis (SDS-PAGE). For the MBP pull-down assays (Fig. 6), purified proteins were mixed at a final concentration of 2.0 μM with 500 nM constitutively active PLK1(T194D) in kinase buffer for 1 hour at 23°C. Reaction mixtures were then mixed with 2 μg of anti-MBP antibodies (Abcam) in lysis buffer and incubated for 2 hours at 4°C. The final concentration of each purified SPD-5 fragment was 75 nM. After the incubation, the proteins were incubated with protein A beads (Thermo Fisher Scientific, no. 88845) for 1 hour at 4°C. The beads were washed five times with lysis buffer and resuspended in sample buffer prior to analysis on SDS-PAGE. The number of times each fragment was analyzed is listed in table S4.

### Gel filtration assay

Purified proteins were concentrated as necessary using 10 kDa MWCO Amicon Ultra concentrators (Millipore) and subjected to SEC on a Superose 6 increase 10/300 or a Superdex 200 increase 10/300 gel filtration column (Cytiva) equilibrated in gel filtration buffer [20 mM tris-HCl (pH 7.5), 50 mM NaCl, 5 mM EGTA, 2 mM MgCl_2_, and 1 mM DTT]. Peak fractions were collected, reconcentrated using 10 kDa MWCO Amicon Ultra concentrators (Millipore), and analyzed by SDS-PAGE and Coomassie staining. To assess whether there was a shift in native molecular weight or hydrodynamic radius of SPD-5 N fragments after PLK1 treatment, 25 μM of purified proteins by gel filtration was mixed with or without 1 μM of constitutive active PLK1-6His in the gel filtration buffer containing 5 mM MgCl_2_ and 0.2 mM ATP, and incubated for 1 hour at 23°C. After the incubation, the reaction mixture was subjected to SEC on Superose 6 increase 10/300 (Cytiva) gel filtration column to analyze their hydrodynamic radius. To estimate hydrodynamic radius, thyroglobulin (2 mg/ml; Stroke’s radius: 85.0 Å), ferritin (0.3 mg/ml; 61.0 Å), aldolase (3 mg/ml; 48.1 Å), and ovalbumin (4 mg/ml; 30.5 Å) were used as standards. The number of times each fragment was analyzed is listed in table S4.

### Multiangle light scattering analysis

Prior to analysis of purified proteins, a Superose 6 increase 10/300 or Superdex 200 increase 10/300 gel filtration column (Cytiva) was equilibrated with gel filtration buffer overnight at room temperature. A total of 120 μl of each purified protein (~2.0 mg/ml) was autoloaded onto the column. Elution and light scattering of the tested proteins were monitored using Wyatt Technology HPLC (high-performance liquid chromatography) software. After each run, baselines of ultraviolet absorbance and light scattering were established, and protein peaks were defined to obtain the predicted molecular weights. SEC-MALS data were exported and graphed using GraphPad Prism software. The number of times each fragment was analyzed is listed in table S4.
