# Supplementary material for: Phosphorylation remodels the mitotic centrosome matrix to generate bipartite γ-tubulin complex docking sites
Source: Sci Adv. 2026 May 27;12(22):eaed6539. doi: 10.1126/sciadv.aed6539 (PMC13215203; doi:10.1126/sciadv.aed6539)
Supplement: Supplementary file 1 — Figs. S1 to S3 Tables S1 to S4 [file sciadv.aed6539_sm.pdf]

Science Advances
AAAS

Supplementary Materials for

Phosphorylation remodels the mitotic centrosome matrix to generate bipartite γ-tubulin complex docking sites

Midori Ohta *et al.*

Corresponding author: Midori Ohta, midori.ota@oist.jp; Karen Oegema, koegema@ucsd.edu

Sci. Adv. **12**, eaed6539 (2026)
DOI: 10.1126/sciadv.aed6539

This PDF file includes:

Figs. S1 to S3
Tables S1 to S4

Figure S1

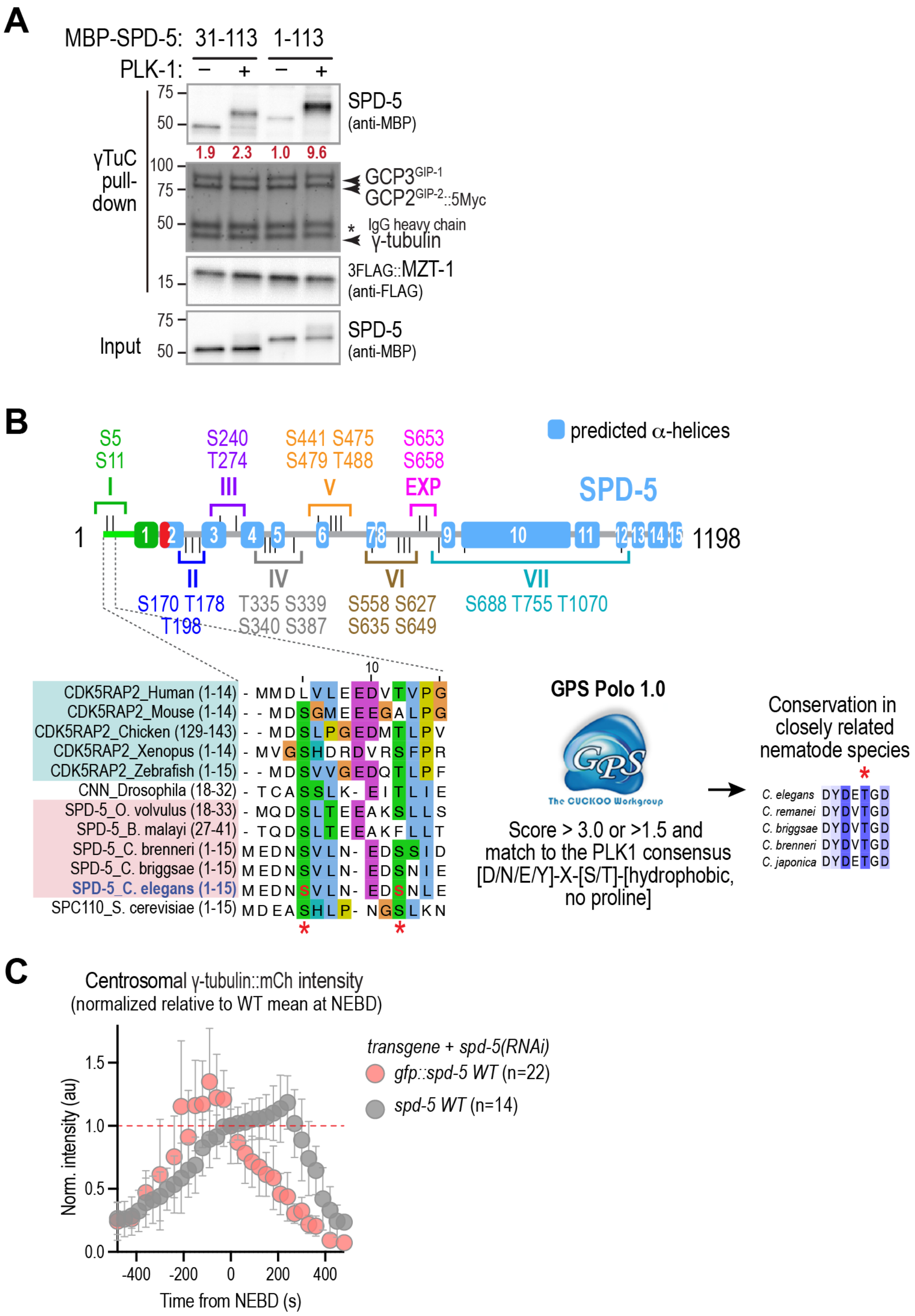
A
MBP-SPD-5: 31-113 1-113
PLK-1: − + − +
γTuC pull-down
SPD-5 (anti-MBP)
1.9 2.3 1.0 9.6
GCP3GIP-1
GCP2GIP-2::5Myc
IgG heavy chain
γ-tubulin
3FLAG::MZT-1 (anti-FLAG)
Input
SPD-5 (anti-MBP)
B
predicted α-helices
SPD-5
S5 S11
I
S240 T274
III
S441 S475 S479 T488
V
S653 S658
EXP
II
S170 T178 T198
IV
T335 S339 S340 S387
VI
S558 S627 S635 S649
VII
S688 T755 T1070
1
1198
CDK5RAP2_Human (1-14)
CDK5RAP2_Mouse (1-14)
CDK5RAP2_Chicken (129-143)
CDK5RAP2_Xenopus (1-14)
CDK5RAP2_Zebrafish (1-15)
CNN_Drosophila (18-32)
SPD-5_O. volvulus (18-33)
SPD-5_B. malayi (27-41)
SPD-5_C. brenneri (1-15)
SPD-5_C. briggsae (1-15)
SPD-5_C. elegans (1-15)
SPC110_S. cerevisiae (1-15)
GPS Polo 1.0
The CUCKOO Workgroup
Score > 3.0 or >1.5 and match to the PLK1 consensus [D/N/E/Y]-X-[S/T]-[hydrophobic, no proline]
Conservation in closely related nematode species
C. elegans DYDETGD
C. remanei DYDVTGD
C. briggsae DYDVTGD
C. brenneri DYDVTGD
C. japonica DYDETGD
C
Centrosomal γ-tubulin::mCh intensity (normalized relative to WT mean at NEBD)
Norm. intensity (au)
Time from NEBD (s)
transgene + spd-5(RNAi)
gfp::spd-5 WT (n=22)
spd-5 WT (n=14)

Fig. S1. Putative PLK1 target sites in PRGB1 and their sequence alignment. **(A)** Binding assays, conducted as outlined in Fig. 1C, with γTuC-coated beads and MBP-6His-HA (M6HH)-tagged SPD-5 fragments, preincubated with or without PLK1 as indicated. SPD-5 and MZT-1 were analyzed by immunoblotting using the indicated antibodies; γ-tubulin, GCP2^{GIP-2}, and GCP3^{GIP-1} were detected by Coomassie staining. Numbers below the SPD-5 fragment bands indicate band intensity relative to SPD-5 1-113 in the absence of PLK1 phosphorylation. Asterisk indicates the IgG heavy chain of the anti-Myc antibody used for the Myc IP. The blot for SPD-5 1-113 is the same as that used in Fig. 1E. **(B)** Top: Schematic depicting candidate PLK1 sites identified using the method shown in the lower right that were mutated in the indicated regional clusters in a prior study that identified cluster II as causing penetrant embryonic lethality (*38*). Bottom Left: Alignment of the indicated sequences from the N-termini of CDK5RAP2 family proteins across species, highlighting potential conservation of the S5 and S11 sites. Phosphorylation sites in alignments are marked with red asterisks. (**C**) Quantification of centrosomal fluorescence intensity over time for γ-tubulin::mCherry in the presence of untagged (*grey*; data reproduced from (*38*)) or GFP-tagged (*red*) SPD-5 after depletion of endogenous SPD-5 by RNAi. Centrosomal fluorescence was normalized by dividing by the mean at nuclear envelope breakdown (NEBD) in each condition (marked with a red dashed line). Error bars are the standard deviation (SD). *n* is the number of centrosomes imaged from at least five biological replicates for each condition.

Figure S2

A

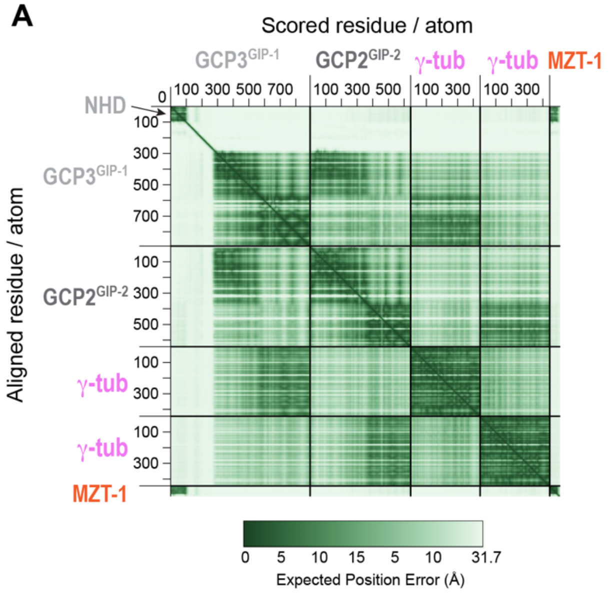
Scored residue / atom
GCP3GIP-1
GCP2GIP-2
γ-tub
γ-tub
MZT-1
NHD
Aligned residue / atom
0
100
300
500
700
Expected Position Error (Å)
0 5 10 15 5 10 31.7

B

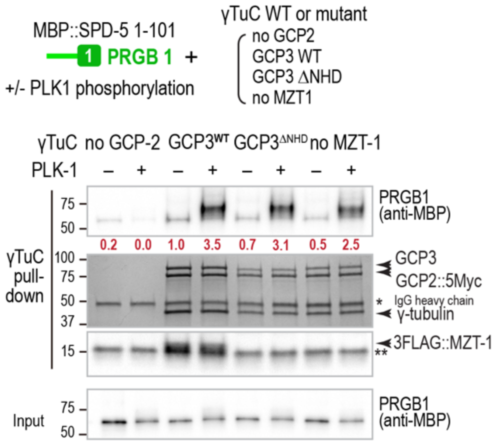
MBP::SPD-5 1-101
1 PRGB 1 +
+/- PLK1 phosphorylation
γTuC WT or mutant
no GCP2
GCP3 WT
GCP3 ΔNHD
no MZT1
γTuC no GCP-2 GCP3WT GCP3ΔNHD no MZT-1
PLK-1 – + – + – + – +
PRGB1 (anti-MBP)
0.2 0.0 1.0 3.5 0.7 3.1 0.5 2.5
γTuC pull-down
GCP3
GCP2::5Myc
* IgG heavy chain
γ-tubulin
3FLAG::MZT-1
**
Input
PRGB1 (anti-MBP)
100
75
50
37
15

Fig. S2. MZT-1 is dispensable for PRGB1 binding to γTuC. (A) Predicted Aligned Error (PAE) plot generated using PAEViewer (*74*) for the *C. elegans* γTuC model in *Fig. 4A*. The MZT-1–GCP3$^{GIP\text{-}1}$ NHD module is separated by a disordered linker and is not positioned relative to the core heterotetrameric complex. **(B)** Expanded version of the western blots/coomassie gel shown in *Fig. 4C* that includes controls in which the plasmids encoding GCP2 which has the Myc tag for the IP (*left two lanes*) or MZT-1 (right two lanes) were omitted during γTuC assembly. Like deletion of the GCP3$^{GIP\text{-}1}$ NHD, assembling the γTuC without MZT-1 also does not impact its PLK1-dependent binding to PRGB1. Beads prepared with the indicated γTuC components were incubated with MBP-6His-HA-tagged SPD-5 aa 1-101 after preincubation with or without PLK1 as indicated. SPD-5 and MZT-1 were analyzed by immunoblotting using the indicated antibodies; γ-tubulin, GCP2$^{GIP\text{-}2}$, and GCP3$^{GIP\text{-}1}$ were detected by Coomassie staining. Numbers below the PRGB1 bands indicate band intensity relative to PRGB1 pulled down by γTuC assembled in the presence of WT GCP3 in the absence of PLK1 phosphorylation. Single asterisk indicates the IgG heavy chain of the anti-Myc antibody used for the Myc IP. Double asterisk marks the location of a non-specific band.

Figure S3

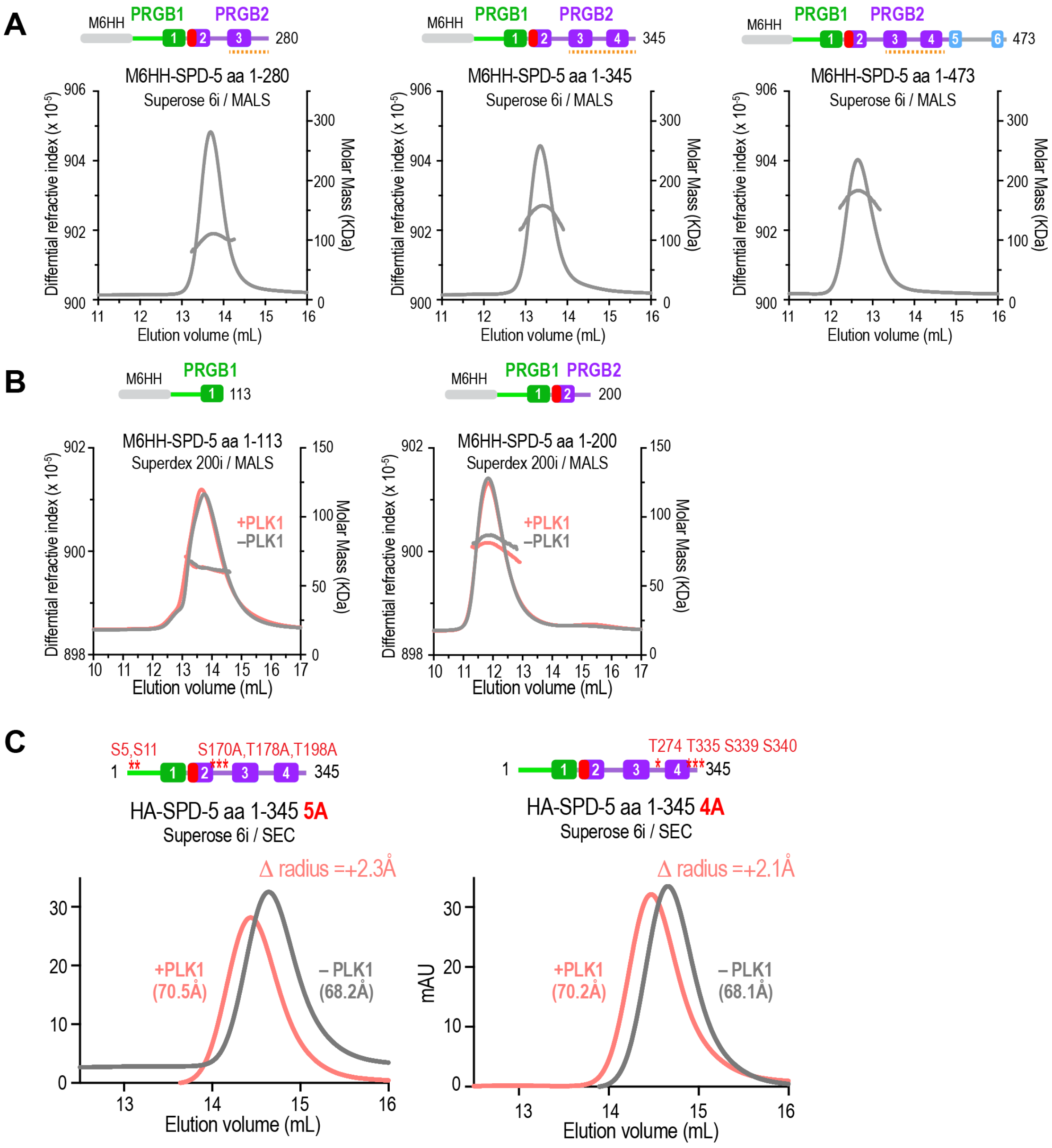
A
M6HH
PRGB1
PRGB2
280
M6HH-SPD-5 aa 1-280
Superose 6i / MALS
345
M6HH-SPD-5 aa 1-345
473
M6HH-SPD-5 aa 1-473
Differential refractive index (x 10⁻⁵)
Molar Mass (KDa)
Elution volume (mL)
B
113
M6HH-SPD-5 aa 1-113
Superdex 200i / MALS
200
M6HH-SPD-5 aa 1-200
+PLK1
–PLK1
C
S5,S11
S170A,T178A,T198A
HA-SPD-5 aa 1-345 5A
Superose 6i / SEC
Δ radius =+2.3Å
+PLK1 (70.5Å)
– PLK1 (68.2Å)
T274 T335 S339 S340
HA-SPD-5 aa 1-345 4A
Δ radius =+2.1Å
+PLK1 (70.2Å)
– PLK1 (68.1Å)
mAU
Elution volume (mL)

Fig. S3. The SPD-5 N-terminus undergoes a PLK1-stimulated conformational change as the result of distributed phosphorylation across the N-terminus. (A) SEC-MALS data for MBP-6His-HA-tagged (M6HH)-tagged SPD-5 aa 1-280, aa 1-345, and aa 1-473 used to determine the values reported in Fig. 5A. **(B)** SEC-MALS data for MBP-6His-HA-tagged (M6HH)-tagged SPD-5 aa 1-113 and SPD-5 aa 1-200 before and after PLK1 phosphorylation used to determine the values in Fig. 5A. The two fragments are monomeric both in the presence and absence of PLK1 phosphorylation and do not exhibit a phosphorylation-dependent shift in elution volume. Orange dashed line marks the region required for dimerization. **(C)** Size exclusion chromatography (SEC) analysis of HA-tagged SPD-5 aa 1-345 with the indicated sets of predicted PLK1 sites mutated to alanine after preincubation with or without PLK1 as indicated. The hydrodynamic radii of the SPD-5 fragments were calculated based on standard proteins and are shown in parentheses and the change in radius induced by phosphorylation is in the upper right corner of each graph.

Table S1. ***C. elegans*** **strains used in this study.**

Strain #	Genotype	Figure
OD4412	ltSi569[oxTi185; pOD1110/pSW008; CEOP3608 TBG-1::mCherry; cb-unc-119(+)]I; ltSi1216 [pOD1021/pVV103; Pspd-2::GFP::SPD-5 reencoded; cb-unc-119(+)]II; unc-119(ed3) III	2A-C, 3B, S1C
MOW11	ltSi569[oxTi185; pOD1110/pSW008; CEOP3608 TBG-1::mCherry; cb-unc-119(+)]I; ltSi1967 [pMO1326; Pspd-2::GFP::SPD-5 reencoded (deletion aa61-93)::spd-5 3'UTR; cb-unc-119(+)]II	2A
OD5196	ltSi569[oxTi185; pOD1110/pSW008; CEOP3608 TBG-1::mCherry; cb-unc-119(+)]I; ltSi1532 [pMO91; Pspd-2::gfp::spd-5 S5A/S11A::spd-5 3'UTR; cb-unc119(+)]II; unc-119(ed3) III	2B
OD5220	ltSi569[oxTi185; pOD1110/pSW008; CEOP3608 TBG-1::mCherry; cb-unc-119(+)]I; ltSi1647 [pMO253; Pspd-2::GFP::SPD-5 aa61-1198 reencoded; cb-unc-119(+)]II; unc-119(ed3) III	2B
MOW09	ltSi569[oxTi185; pOD1110/pSW008; CEOP3608 TBG-1::mCherry; cb-unc-119(+)]I; ltSi1704 [pMO269; Pspd-2::GFP::SPD-5 aa264-1198 reencoded; cb-unc-119(+)]II; unc-119(ed3) III	2C
MOW21	ltSi569[oxTi185; pOD1110/pSW008; CEOP3608 TBG-1::mCherry; cb-unc-119(+)]I; ltSi2010 [pMO1503; Pspd-2::GFP::SPD-5 reencoded (deletion aa135-180)::spd-5 3'UTR; cb-unc-119(+)]II; unc-119(ed3)III (#18)	3B
OD4211	ltSi1216[pOD1021/pVV103; Pspd-2::GFP::SPD-5 reencoded; cb-unc-119(+)]II; unc-119(ed3) III	2D
OD5160	ltSi1641 [pMO252; Pspd-2::GFP::SPD-5 aa114-1198 reencoded; cb-unc-119(+)]II; unc-119(ed3) III	2D
OD5262	ltSi1704[pMO269; Pspd-2::GFP::SPD-5 aa264-1198 reencoded; cb-unc-119(+)]II; unc-119(ed3) III	2D
OD4840	ltSi569[oxTi185; pOD1110/pSW008; CEOP3608 TBG-1::mCherry; cb-unc-119(+)]I; ltSi1129[pZZ2; Pspd-2::SPD-5 (re-encoded);cb-unc-119(+)]II; unc-119(ed3)III	S1C

Table S2. Oligos used for dsRNA production.

Gene	Oligonucleotide 1 (5' → 3')	Oligonucleotide 2 (5' → 3')	Template	Concentration (mg/mL)
spd-5 (F56A3.4)	TAATACGACTCACTATAGGTGGAATTGTCCGCTACTGAT	AATTAACCCTCACTAAAGGTGTATTCAACGAGTGCCTGA	N2 genomic DNA	1.3 - 1.4

Table S3. Plasmids used in this study.

plasmid #	Description	Bacterial selection
**pMO97**	p3XFLAG-CMV-7.1	Ampicillin
**pMO98**	SP254_CS2P_mtc2	Ampicillin
**pMO103**	pCMV-TBG-1 (*C. elegans* γ-tubulin)	Ampicillin
**pMO106**	pCMV-GIP-2-5Myc	Ampicillin
**pMO113**	pCMV-GIP-1	Ampicillin
**pMO135**	pCMV-3FLAG-MZT-1	Ampicillin
**pMO133**	pGEX-6P-1-GST-SPD-5 aa1-473	Ampicillin
**pMO193**	pGEX-6P-1-GST-SPD-5 aa181-473	Ampicillin
**pMO205**	pGEX-6P-1-GST-SPD-5 aa101-473	Ampicillin
**pMO208**	pGEX-6P-1-GST-SPD-5 aa135-473	Ampicillin
**pMO245**	pGEX-6P-1-GST-HA-SPD-5 aa1-270	Ampicillin
**pMO247**	pGEX-6P-1-GST-HA-SPD-5 aa1-345	Ampicillin
**pMO233**	pGEX-6P-1-GST-HA-SPD-5 aa1-473	Ampicillin
**pMO258**	pMAL-His6-TEV-HA-SPD-5 aa1-113	Ampicillin
**pMO260**	pMAL-His6-TEV-HA-SPD-5 aa1-200	Ampicillin
**pMO263**	pMAL-His6-TEV-HA-SPD-5 aa1-280	Ampicillin
**pMO264**	pMAL-His6-TEV-HA-SPD-5 aa59-113	Ampicillin
**pMO285**	pMAL-His6-TEV-HA-SPD-5 aa1-345	Ampicillin
**pMO287**	pMAL-His6-TEV-HA-SPD-5 aa1-137	Ampicillin
**pMO288**	pMAL-His6-TEV-HA-SPD-5 aa1-170	Ampicillin
**pMO290**	pGEX-6P-1-GST-HA-SPD-5 aa1-113 S5A S11A	Ampicillin
**pMO292**	pMAL-His6-TEV-HA-SPD-5 aa1-473	Ampicillin
**pMO293**	pMAL-His6-TEV-HA-SPD-5 aa31-113	Ampicillin
**pMO302**	pMAL-His6-TEV-HA-SPD-5 aa1-101	Ampicillin
**pMO303**	pMAL-His6-TEV-HA-SPD-5 aa1-60	Ampicillin
**pMO306**	pGEX-6P-1-GST-HA-SPD-5 aa1-345 S5A S11A S170A T178A T198A	Ampicillin
**pMO307**	pGEX-6P-1-GST-HA-SPD-5 aa1-345 T274A T335A S339A S340A	Ampicillin
**pMO308**	pGEX-6P-1-GST-HA-SPD-5 aa1-345 S5A S11A S170A T178A T198A T274A T335A S339A S340A	Ampicillin
**pMO1323**	pCMV-GIP-1 delta NHD	Ampicillin
**pMO1358**	pMAL-His6-TEV-HA-SPD-5 aa135-200	Ampicillin
**pMO1360**	pMAL-His6-TEV-HA-SPD-5 aa135-280	Ampicillin
**pMO1362**	pMAL-His6-TEV-HA-SPD-5 aa135-345	Ampicillin

**pMO1368**	pGEX-6P-1-GST-SPD-5 aa135-473 S170A T178A T198A	Ampicillin
**pMO1370**	pGEX-6P-1-GST-SPD-5 aa135-473 T274A T335A S339A S340A	Ampicillin
**pMO1372**	pGEX-6P-1-GST-SPD-5 aa135-473 S170A T178A T198A T274A T335A S339A S340A	Ampicillin
**pMO1389**	pHalo-His6-TEV-HA-SPD-5 aa135-200	Ampicillin
**pMO1401**	pHalo-His6-TEV-HA-SPD-5 aa135-345	Ampicillin
**pMO1402**	pHalo-His6-TEV-HA-SPD-5 aa200-345	Ampicillin
**pMO1404**	pHalo-His6-TEV-HA-SPD-5 aa260-345	Ampicillin
**pMO1670**	pMAL-His6-TEV-HA-SPD-5 aa1-263	Ampicillin

Table S4. Experiment Replication

Experiment	Sample	Number of replicates	Figures
γTuC pull-down assays	MBP-6His-HA-SPD-5 1-113	≥ 5 biological replicates	1E, 1F, 1I
	MBP-6His-HA-SPD-5 1-137	once	1E
	MBP-6His-HA-SPD-5 1-170	once	1E
	MBP-6His-HA-SPD-5 1-200	≥ 5 biological replicates	1E
	MBP-6His-HA-SPD-5 1-60	once	1F
	MBP-6His-HA-SPD-5 1-101	≥ 3 biological replicates	1F, 4C
	MBP-6His-HA-SPD-5 59-113	≥ 3 biological replicates	1F
	HA-SPD-5 1-270	≥ 2 biological replicates	1G
	HA-SPD-5 1-345	≥ 2 biological replicates	1G
	HA-SPD-5 1-473	≥ 2 biological replicates	1G
	MBP-6His-HA-SPD-5 1-113 S5A S11A	once	1F
	SPD-5 1-473	≥ 5 biological replicates	3A, 4D
	SPD-5 101-473	≥ 3 biological replicates	3A
	SPD-5 135-473	≥ 3 biological replicates	3A, 3E
	SPD-5 181-473	≥ 3 biological replicates	3A
	MBP or Halo-HA -SPD-5 135-200	≥ 3 biological replicates	3C, 5B
	MBP or Halo-HA -SPD-5 135-280	once	5B
	MBP or Halo-HA -SPD-5 135-345	≥ 3 biological replicates	3C,4C, 5B
	MBP or Halo-HA -SPD-5 200-345	once	3C
	MBP or Halo-HA -SPD-5 260-345	once	3C
	SPD-5 135-473 7A	≥ 2 biological replicates	3E
	SPD-5 135-473 3A	once	3E
	SPD-5 135-473 4A	once	3E
	no GCP-2/GCP3 FL	≥ 3 biological replicates	4B
	GCP-2-5Myc/GCP3 FL	≥ 3 biological replicates	4B
	GCP-2-5Myc/GCP3 ΔNHD	≥ 3 biological replicates	4B
	MBP-6His-HA-SPD-5 1-101 vs GCP3 FL w/o GCP2	≥ 2 biological replicates	S2B
	MBP-6His-HA-SPD-5 1-101 vs GCP3 FL with GCP2	≥ 3 biological replicates	4C, S2B
	MBP-6His-HA-SPD-5 1-101 vs GCP3 ΔNHD with GCP2	≥ 2 biological replicates	4C, S2B
	MBP-6His-HA-SPD-5 1-101 vs GCP3 FL w/o MZT-1	≥ 2 biological replicates	S2B
	MBP-6His-HA-SPD-5 135-345 vs GCP3 FL	≥ 2 biological replicates	4C

	MBP-6His-HA-SPD-5 135-345 vs GCP3 ΔNHD	≥ 2 biological replicates	4C
	SPD-5 1-473 vs GCP3 ΔNHD	≥ 2 biological replicates	4D
	MBP-6His-HA-SPD-5 30-113	once	S1A
MBP pull-down assays	HA-SPD-5 101-473 vs MBP-6His-HA-SPD-5 1-60	≥ 2 biological replicates	6A
	HA-SPD-5 101-473 vs MBP-6His-HA-SPD-5 1-101	≥ 2 biological replicates	6A
	MBP-6His-HA-SPD-5 1-113 vs Halo-SPD-5 135-200	once	6B
	MBP-6His-HA-SPD-5 1-113 vs Halo-SPD-5 200-345	once	6B
SEC-MALS	MBP-6His-HA or untagged SPD-5 1-473 without PLK-1	≥ 3 biological replicates	5A, S3A
	SPD-5 1-473 with PLK-1	≥ 2 biological replicates	5A, S3A
	MBP-6His-HA SPD-5 1-113 without PLK-1	≥ 3 biological replicates	5A, S3B
	MBP-6His-HA SPD-5 1-113 with PLK-1	≥ 2 biological replicates	5A, S3B
	MBP-6His-HA SPD-5 1-200 without PLK-1	≥ 3 biological replicates	5A, S3B
	MBP-6His-HA SPD-5 1-200 with PLK-1	≥ 2 biological replicates	5A, S3B
	MBP-6His-HA SPD-5 1-280 without PLK-1	once	5A, S3A
	MBP-6His-HA SPD-5 1-345 without PLK-1	once	5A, S3A
SEC	HA-SPD-5 1-345 WT	≥ 2 biological replicates	5C
	HA-SPD-5 1-345 9A	once	5C
	HA-SPD-5 135-345	once	5D
	HA-SPD-5 1-345 4A	once	S3C
	HA-SPD-5 1-345 5A	once	S3C
